# Improved One-Class Modeling of High-Dimensional Metabolomics Data via Eigenvalue-Shrinkage

**DOI:** 10.3390/metabo11040237

**Published:** 2021-04-13

**Authors:** Alberto Brini, Vahe Avagyan, Ric C. H. de Vos, Jack H. Vossen, Edwin R. van den Heuvel, Jasper Engel

**Affiliations:** 1 Department of Mathematics and Computer Science, Eindhoven University of Technology, 5600 MB Eindhoven, The Netherlands; e.r.v.d.heuvel@tue.nl; 2 Biometris, Wageningen University and Research, Droevendaalsesteeg 1, 6708 PB Wageningen, The Netherlands; vahe.avagyan@wur.nl (V.A.); jasper.engel@wur.nl (J.E.); 3 Bioscience, Wageningen University and Research, Droevendaalsesteeg 1, 6700 AA Wageningen, The Netherlands; ric.devos@wur.nl; 4 Plant Breeding, Wageningen University and Research, Droevendaalsesteeg 1, 6700 AJ Wageningen, The Netherlands; jack.vossen@wur.nl

**Keywords:** high-dimensional data, one-class model, untargeted metabolomics, mahalonobis distance, eigenvalue-shrinkage, critical value, scaled Chi-square distribution

## Abstract

One-class modelling is a useful approach in metabolomics for the untargeted detection of abnormal metabolite profiles, when information from a set of reference observations is available to model “normal” or baseline metabolite profiles. Such outlying profiles are typically identified by comparing the distance between an observation and the reference class to a critical limit. Often, multivariate distance measures such as the Mahalanobis distance (MD) or principal component-based measures are used. These approaches, however, are either not applicable to untargeted metabolomics data, or their results are unreliable. In this paper, five distance measures for one-class modeling in untargeted metabolites are proposed. They are based on a combination of the MD and five so-called eigenvalue-shrinkage estimators of the covariance matrix of the reference class. A simple cross-validation procedure is proposed to set the critical limit for outlier detection. Simulation studies are used to identify which distance measure provides the best performance for one-class modeling, in terms of type I error and power to identify abnormal metabolite profiles. Empirical evidence demonstrates that this method has better type I error (false positive rate) and improved outlier detection power than the standard (principal component-based) one-class models. The method is illustrated by its application to liquid chromatography coupled to mass spectrometry (LC-MS) and nuclear magnetic response spectroscopy (NMR) untargeted metabolomics data from two studies on food safety assessment and diagnosis of rare diseases, respectively.

## 1. Introduction

Due to the rapid advances in comprehensive untargeted metabolomics, generating relative abundance values of hundreds to thousand metabolites present in crude extracts, it is possible to examine the metabolite profile of an individual observation in unprecedented detail. Analytical techniques such as liquid chromatography coupled to mass spectrometry (LC-MS) or nuclear magnetic response spectroscopy (NMR) allow for the global and unbiased profiling of a wide range of metabolites in a single biological sample. Although these metabolites are detected in an untargeted fashion, a targeted statistical approach is commonly used to interrogate the acquired data. Typically, cases (i.e., tests) and controls (i.e., references) are compared as a two-class classification problem using, for example, partial least squares—discriminant analysis (PLS-DA). However, this approach is often unsuitable because the classes are not well defined due to associated populations being biologically too heterogeneous; or when data from a class is difficult or expensive to obtain, resulting in a too low amount of samples; or when an observation being classified does not belong to any of the predefined classes (i.e., an unknown or unexpected effect).

In one-class modeling, the focus lies on the classification of observations to a single, specific class, which we will refer to as the reference class [1]. In particular, data from a set of reference observations (a random sample from the reference class) is used to characterize the normal range of metabolic profiles of that class. Deviations in the metabolite profile of new observations compared to this normal range then provide an untargeted signal indicative of an outlier (abnormal observation). Here, untargeted refers to the fact that a multitude of outliers, whose metabolite profiles may differ from the reference class in various ways, can be detected by the same one-class classifier. This way, the issues of multi-class classification mentioned above are circumvented. Applications of one-class classification in combination with untargeted metabolomics include food authentication, health monitoring, and risk assessment of genetically modified (GM) crops [2,3,4]. In food authentication, the reference class is formed by the product of interest [2]. Outliers potentially correspond to adulterants. In health monitoring, the one-class classifier is used for disease detection [3]. The model is trained by metabolite data from a set of healthy references. Several methods have been proposed to identify the metabolites associated to an observation being an outlier from the one class, i.e., disease diagnosis [5]. The approach was used for the diagnosis of rare diseases, where it may be difficult to obtain observations from a specific disease [3]. It has also been proposed for detection of individual responses to treatment of Hepatocarcinogenesis [6]. In safety assessment of GM crops, the reference class consists of varieties that have a history of safe use for human consumption [4]. An outlier may be indicative of an undesired effect in the GM event, as a result of the introduced trait or of the GM method that may pose a hazard. Note that outlier detection may be a useful element in assessing food safety, but is not in itself sufficient to show the equivalence of the genetically modified and reference crops, because the absence of a proof is not a proof of absence (i.e., a proof of safety) [7]. Another potential application of one-class modeling in metabolomics would be quality control of a mass-spectrometer over time [8,9]. 

The Mahalanobis Distance (MD) is a multivariate generalization for measuring how many standard deviations an observation is away from the center of a class. The MD is often used in one-class modeling, where it is assumed that the distribution of the reference class is multivariate normal [10]. Observations with a MD above a critical limit are marked as an outlier. The MD, however, was developed for traditional data sets where the number of observations (N) is much larger than the number of variables or metabolites (P). The measure becomes unreliable when N~P and completely breaks down when N≪P [11]. In other words, the MD is not applicable to a typical untargeted metabolomics data set. To circumvent this issue, the dimension of the data is often first reduced by principal component analysis (PCA). Subsequently, the MD (and other distance measures) can be used to detect outliers in the subspace spanned by the principal components and its orthogonal complement. This strategy is used, for example, in Soft Independent Modeling by Class Analogy (SIMCA) [12]. However, we will show in Section 2 that such popular PCA-based outlier detection criteria also have serious drawbacks when applied to high-dimensional data (N≪P), including metabolomics data. Other authors have reported similar issues in these settings [12,13]. In particular, the type I error of PCA-based one-class models is not controlled well, i.e., too many reference observations are incorrectly flagged as outliers. In addition, the performance of a PCA-based one-class model critically depends on the number of selected principal components, which is not straightforward to choose in practice. Finally, as a consequence of the dimension reduction step, outliers cannot be identified on the basis of a single distance metric.

**Figure 1:**
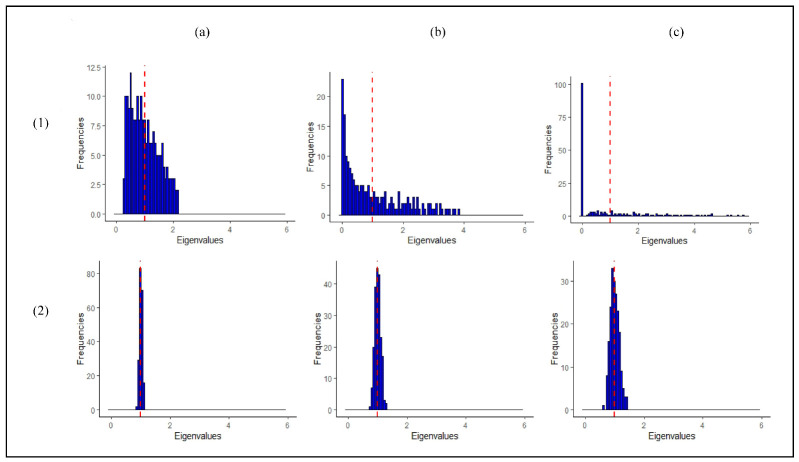
Estimates of the eigenvalues of the covariance matrix of the reference class for (**a**) 800, (**b**) 200, and (**c**) 100 reference observations and 200 variables (metabolites). The top and bottom rows correspond to the sample and (“L LW”) eigenvalue-shrinkage estimates of the covariance matrix, respectively. The data are i.i.d. samples from N(0, Σ) with population covariance matrix Σ=I, i.e., the population eigenvalues are all equal to one as is indicated by the vertical red dashed line. In line with the simulation settings performed in rest of the paper, the data is auto-scaled. Note that, in particular, the dispersion of the sample eigenvalue estimates around the population value increases when the number of observations decreases. This result is generally observed and not restricted to the case shown here [14].

The estimate of the MD critically depends on the estimate of the covariance matrix of the reference class, which describes the variation in the metabolite profiles of the references around the mean (of the references). In particular, the eigenvalues of the covariance matrix correspond to the variance of the reference observations along different directions (principal components) in a multivariate space. Typically, the well-known sample estimator of the covariance matrix is used to estimate these. As shown in the top row of Figure 1, however, this has several drawbacks for datasets where the number of observations is (much) smaller than the number of variables, such as in untargeted metabolomics data. The variance of the reference observations is overestimated along some directions in multivariate space and underestimated along others. Clearly, this hampers outlier detection, see Supplementary Figure S1. In particular, as shown in Figure 1c, some variance estimates are equal to zero when the number of metabolites is larger than the number of reference observations, and the calculation of the MD completely breaks down in these cases.

As shown in the bottom row in Figure 1, the drawbacks of the sample covariance estimator mentioned above can be overcome by application of a so-called eigenvalue-shrinkage estimator. Briefly, eigenvalue-shrinkage results in a better estimate of the variation between the reference observations. Secondly, this class of methods always returns a so-called positive definite estimate of the covariance matrix. This means that in principle the method can always be employed to calculate the MD, even when P≪N. The use of such shrinkage-based estimators for one-class modelling is not widespread in the literature, especially in metabolomics. Recently, Ullah et al. [8] demonstrated the use of eigenvalueshrinkage-based one-class modeling in targeted metabolomics. The method was successfully applied for quality control of GC-MS data involving 25 metabolites. The present paper focuses on eigenvalueshrinkage for one-class modeling in untargeted metabolomics data involving a much larger number of metabolites. We extend the work of Ullah and coworkers in high-dimensional settings by (1) employing more sophisticated eigenvalue-shrinkage approaches in combination with the MD [15,16,17,18] and (2) using a simple procedure to set the critical limit for the shrinkage MD (for identification of outliers). In (1) five shrinkage estimators are considered, namely the so-called Ledoit and Wolf (LW) linear shrinkage estimator that was used by Ullah et al. [8] and four estimators that improve upon LW. The best (in terms of type I error and computational feasibility) shrinkage estimator is identified by simulation and consequently employed for one-class modeling in untargeted metabolomics. 

The application of the shrinkage MD in one-class modeling requires a critical limit to identify outliers. Specification of this limit is not straightforward because the distribution of the resulting MD under the null hypothesis of no outlier is unknown, due to the eigenvalue-shrinkage. We show, however, that the distribution can be approximated well by a (scaled) χ^2^ distribution. The degrees of freedom and scaling factor are obtained from cross-validation using the reference observations. We provide empirical evidence by simulation that this approach outperforms the method of Ullah et al. [8] in terms of type I error. In short, we introduce an improved protocol for one-class modeling in high-dimensional data based on shrinkage, which will be called as “ES-CM” (a.k.a. eigenvalue-shrinkage class-model) throughout the rest of the paper. 

In the next section, the practical application of ES-CM is illustrated by its application to two case studies. We first focus on risk assessment of GM crop varieties using LC-MS data. Next, diagnosis of inborn errors of metabolism (IEM) on the basis of urine 1H-NMR spectra is considered. Subsequently, the statistical properties of ES-CM are investigated by simulation. The results demonstrate that the method has better type I error (false positive rate) and improved power (to detect actual outlying observations) than the standard PCA-based (SIMCA) one-class classifier, and also improves upon other shrinkage-based one-class models such as the one of Ullah et al. Section 3 discusses the results together with possible directions for future research. Finally, in Section 4 materials, methods, and the main theory of ES-CM are presented. 

## 2. Results

### 2.1. Case Study 1: Compositional Safety Assessment of Tubers from Commercial and Cisgenic Potato Varieties 

Safety assessment of GM crop varieties generally focuses on two aspects: intended effects and potential unintended effects of the genetic modification. In Herman et al. [19], it has been argued that safety of potential unintended effects should be considered in the light of normal crop variation. For this purpose, a comparative analysis is performed, where metabolic profiles of the GM variety are compared to those of reference varieties that have a history of safe use. Recently, comprehensive, untargeted metabolomics in combination with a SIMCA one-class model were proposed for this purpose [4,20]. Here, ES-CM is used to analyze untargeted LC-MS-based metabolomics data of a series of potato tuber samples from both commercial genotypes and cisgenic GM varieties, with each sample consisting of a mix of multiple individual tubers per variety. Thus, for the considered case of cisgenesis, the argument of Herman et al. is even more justified because of the native genes that were introduced in these varieties. The samples that are marked as an outlier by ES-CM contain aberrant metabolite profiles in comparison to the references, and therefore may need further evaluation for risk assessment. 

The present study considers the relative abundance values of 458 metabolites, detected by untargeted high mass resolution LC-MS, across 62 potato tuber samples (see the Supplementary Materials for further details about the potato samples). Each sample represents a different potato genotype or environment. Forty-one of these samples were grown in the same location (Wageningen, The Netherlands) and correspond to conventionally bred consumption (i.e., non-starchy) potato varieties with a history of safe use for human consumption, i.e., a sample of the reference class. They were used to train ES-CM. 

ES-CM employs eigenvalue-shrinkage, providing an estimate of the covariance matrix as a combination of the identity and the sample covariance matrices. The contribution of two components is given by the shrinkage intensity (a number between 0 and 1). The optimal eigenvalue-shrinkage intensity was estimated at 0.599. This means that a considerable amount of shrinkage was used to obtain a better estimate of the variation between the reference varieties in multivariate space. Further technical details and interpretations of the shrinkage intensity are given in Section 4.5. In a leave-one-out cross-validation the classification of each reference sample was investigated by ES-CM. A total of 35 observations were correctly classified as inside the one-class, while 6 observations were incorrectly marked as an outlier, see Figure S2b of the Supplementary Materials. This corresponds to a type I error rate of 12.5%, which is larger than the nominal level of 5% that was used, but within the expected range (the binomial Clopper-Pearson interval ranges from 1.1 to 14%) given the limited sample size in this study. 

Next, the trained ES-CM was used to classify the remaining 21 samples. Eight of these samples correspond to eight cisgenic varieties that were enriched with different late blight resistant genes from crossable species [21]. Three samples correspond to starch varieties that, together with the cisgenic varieties, were grown in the same location as the reference group. The remaining 10 samples are consumption varieties with a history of safe use that were obtained from 2 other locations in comparison to the training data. These varieties are also included in the set that forms the reference class. However, these samples are not references themselves because the samples were obtained from another location.

**Figure 2:**
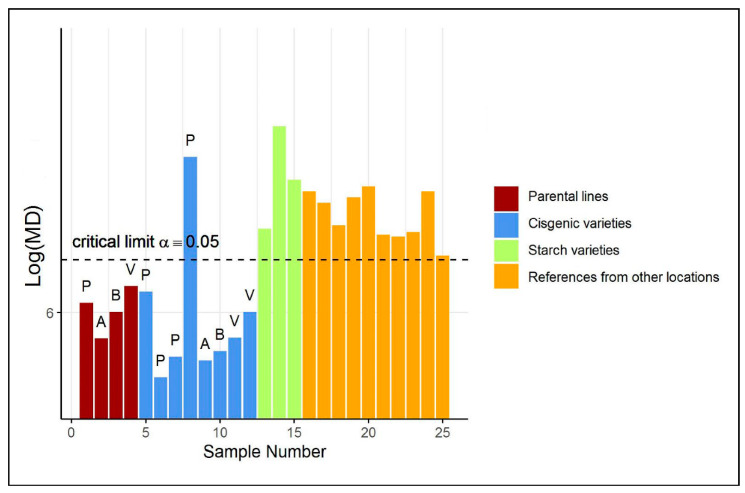
(Shrinkage-based) MD values for the test samples in case study 1, displayed per different groups, in the potato data. For clarity, the 4 parental varieties (“P”, “A”, “B” and “V”) of each cisgenic event are included as well. Note that the 4 values of the parental varieties are based on cross-validated MDs. See Table S10 of the Supplementary Materials for more information on each test sample.

A first investigation based on a Principal Component (PC) score plot (shown in Figure S2a in the Supplementary Materials) of the auto-scaled training and test data does not reveal any abnormal sample in the test group. However, only a relatively low percentage of variance among the reference metabolites is explained by the first two principal components. Arguably, we aim with ES-CM to take all the variation in the data into account. Figure 2 illustrates the classification of the potato tubers included in the test set by ES-CM. The y-axis shows the eigenvalue-shrinkage based MD of each observation. The horizontal dotted line corresponds to the critical limit. The critical limit was determined by cross-validation, see Section 4.6. Any observation with a MD larger than the critical limit is classified as an outlier, i.e., outside the one-class. The cisgenic varieties (in blue) appear to behave differently than the other two test groups (i.e., starch and reference varieties, respectively, in green and orange), as their MD is much closer to those of the parental varieties (in dark red), which are part of the reference group. In fact, the majority of the cisgenic events (7/8) were not flagged as “outliers”. This offers the first indication that cisgenic breeding does not cause a disturbance of global metabolite composition and is therefore equally safe as classical breeding. Furthermore, this is in line with current expectations that the adopted GM modification methods are not thought to create greatly different metabolic phenotypes [4]. The GM cisgenic event that is marked as an outlier (outside the one-class) may well be safe but will require further risk assessment to confirm its safety. A starting point for this could be the identification of the variables (metabolites) that contributed most to this observation being an outlier. Such a procedure is discussed in the next case study. Next, the metabolite profiles of the starchy potatoes were found to be clearly different from the profiles of the reference class. In fact, the reference class contains non-starchy potato varieties only. Therefore, the evaluation of starchy potato varieties by ES-CM is expected to highlight their abnormality. Similarly, all reference varieties from another location were also marked as outlying. This is attributed to the fact that the reference class was defined for samples from location A (see Supplementary Materials), while these samples were grown in different locations, B and C. Thus, the model correctly indicates that these are not part of the reference class. These differences are attributed to environmental variation.

### 2.2. Case Study 2: Diagnosis of Inborn Error of Metabolism 

Recently, a PCA-based one-class model was proposed for disease detection, and, in a subsequent step, identification of the disease biomarker at the level of the individual patient [22]. In this section, ES-CM is applied to identify observations with an abnormal metabolite profile (disease detection). Subsequently, the variables contributing to the observation are identified, which is also briefly touched upon. By means of prior knowledge, these abnormal features can be linked to a disease (disease diagnosis). Here, we focus on analysis of urine 1H-NMR spectra. More specifically, urine samples were acquired from 176 healthy controls. In addition, 1H-NMR data of urine samples of 41 test observations is available. Earlier, the data was analyzed according to current clinical practice, namely by visual inspection of the 1H-NMR spectra by a clinical expert and by means of PCA score plots [3]. By means of visual inspection, abnormal patterns (with respect to the reference class of healthy controls) were identified in each of the spectra from the test set. Many of these could not be identified in a PCA scores plot of the complete data matrix, see Supplementary Figure S3a.

For improved detection of abnormal patterns an ES-CM model was fitted to the preprocessed NMR data, which comprised 246 bins (variables). A subset of healthy controls was selected to train ES-CM, i.e., using a random sample. In line with the original study of Engel et al. [3], the Kennard Stone algorithm [23] was used to select a subset of 120 healthy individuals. 

The remaining 56 individuals were used in the model validation discussed below. The first step in fitting the ES-CM model was to obtain an estimate of the covariance matrix of the class of healthy controls by means of eigenvalue-shrinkage. Compared to case study one, less shrinkage was applied because of the larger ratio between the number of training observations and the number of variables in the data set. More specifically, the eigenvalue-shrinkage intensity was estimated at 0.078. This number is close to zero suggesting that not much shrinkage had to be applied to correct for the bias in the sample covariance matrix of the set of references. Note, however, that the sample covariance matrix was not positive definite, meaning that it could not be used (in combination with the MD) to fit one-class model. Due to eigenvalue-shrinkage, a positive definite estimate of the covariance matrix was obtained, and consequently a one-class model could be fitted. Similar to case study 1, we have evaluated the compliance of each training reference observation (out of the 120) to the one-class by leave-one-out cross-validation. Figure S3b of the Supplementary Materials illustrates that only a few references (9) were detected as outliers. This corresponds to a type I error rate of 7.5%, which is within the expected range (the binomial Clopper-Pearson interval ranges from 1.9–10.6%) for this study. 

**Figure 3:**
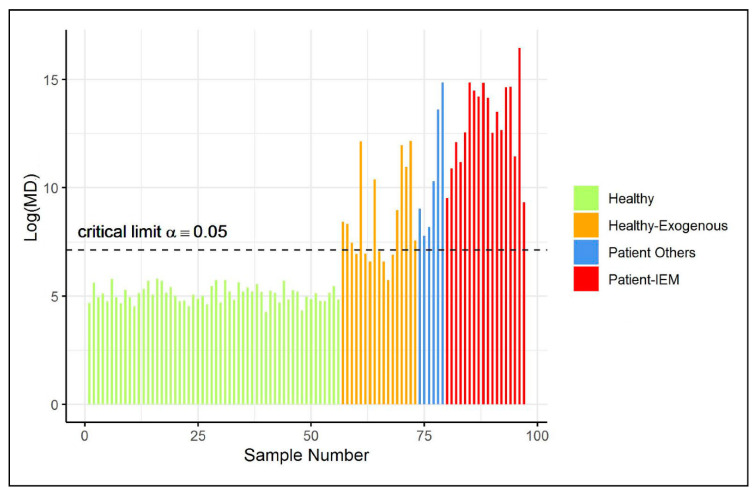
(Shrinkage-based) MD values for test samples in case study 2, displayed per different groups, based on the grouping proposed in Engel et al. [3].

The remaining 56 healthy controls (not included in the training data) were also used to validate the type I error of the ES-CM classifier. Figure 3 illustrates that all of these observations were classified as inside the one-class. Next, 41 additional observations were classified by the fitted ES-CM model. Abnormal patterns (in comparison to the reference class) had earlier been observed in the NMR spectra of these observations by a clinical expert [3]. In other words, this set comprised observations that should be marked as an outlier by ES-CM. More specifically, this test set comprised data from 18 patients who were known to suffer from one of 7 inborn errors of metabolism (IEM), namely (isovaleric) aciduria (1), alkaptonuria (10), cystinuria (2), carboxylase deficiency (1), dehydrogenase deficiency (1), formiminotransferase deficiency (2), and oxoprolinuria (1) (see Engel et al. [3] for more details). Of the remaining 23 samples, 6 are patients who consumed commonly prescribed drugs (i.e., paracetamol, depakine), and the last 17 are healthy individuals with exogenous metabolite profiles due to diet (in comparison to the reference class). Interestingly, Figure 3 shows that all IEM patients were correctly marked as an outlier by ES-CM, highlighting that single ES-CM can be used to detect multiple diseases. Similarly, other patients under medication were also classified outside the class. Most healthy individuals with an exogenous metabolic profile (as defined in Engel et al. [3]) were also detected as abnormal (10/17) with respect to the healthy group of references. For the (7/17) cases classified inside the class, we found characteristic compounds associated to fish (1), artificial sweetener consumptions (3), drug intake (1), and bacteria contamination (2). 

In this context, after an observation is marked as an outlier, it might be beneficial to detect which resonances in the NMR data contributed most to the observation being an outlier. This information could be used to identify the pattern of metabolites for which the patient differs most from the references, i.e., a biomarker at the level of the individual patient [5]. Recently, various approaches were reviewed for this purpose [5]. A brief description is given in the Supplementary Materials. These can easily be combined with the eigenvalue-shrinkage estimator used by ES-CM. Two examples are shown in Table S5 of the Supplementary Materials corresponding to an alkaptonuria patient and a person who consumed paracetamol. 

### 2.3. Synthetic Data Experiments

For reasons described in Section 4.5, 5 shrinkage estimators of the covariance matrix of the reference class were initially considered to be used for one-class modeling. These methods are referred to as “L LW” [14], “L OA” [16], “L T” [15], “nL LW” [24], and “nL vW” [17]. The first 3 methods are so-called linear eigenvalue-shrinkage estimators, while the latter 2 are nonlinear approaches. As explained in Section 4.5, nonlinear shrinkage is typically expected to result in the best estimate of the covariance matrix of the reference class, but linear shrinkage often offers a good approximation while being computationally much more efficient. 

The shrinkage estimators are evaluated by applying them to synthetic data since there is often no clear “ground-truth” in real metabolomics experiments. In the simulation 3 covariance structures of the reference class were considered. First, the eigenvalue-shrinkage estimators were compared to each other with respect to computational speed and type I error of the corresponding one-class model. The best performing estimator was selected for further use in ES-CM, including the case studies described above. Next, the power of the ES-CM to identify outliers was investigated and compared to that of a popular PCA-based one-class model. The complete setup of the synthetic data experiments is outlined in Section 4.3.

#### 2.3.1. Assessment of the Computational Speed 

The computational efficiency of 5 eigenvalue-shrinkage estimators was compared for synthetic data involving N=25 reference observations and P=250 metabolites. More specifically, the time (in seconds) needed for the procedure to estimate the covariance matrix for a single dataset was considered. Table 1 shows that the linear shrinkage estimators are computationally much more efficient compared to the nonlinear methods. In particular, the estimation of the covariance matrix by “nL vW” took more than 8 min. Note that setting the critical limit of ES-CM requires iterative estimation of the covariance matrix in a cross-validation framework. Therefore, from a computational perspective, “nL vW” was not considered a feasible approach, especially for the analysis of untargeted metabolomics data with P≫250. For similar reasons, the application of the other non-linear shrinkage method (“nL LW”) will also often be unfeasible.

#### 2.3.2. Type I Error of the Eigenvalue-Shrinkage Approaches

Next, the type I error of one-class models based on the MD in combination with the 5 eigenvalue-shrinkage methods was investigated. Seemingly, for the single data set considered in Table 1, the type I error of one-class models based on non-linear shrinkage is closer to the nominal level of 5% compared to the linear shrinkage estimators. However, the results of linear shrinkage appeared to be quite close. Given the computational expense of the non-linear methods, subsequent investigations mainly focused on the performance of linear eigenvalue-shrinkage for one-class modeling. For the sake of comparison, the least computationally expensive nonlinear method, “nL LW”, was also included.

**Figure 4:**
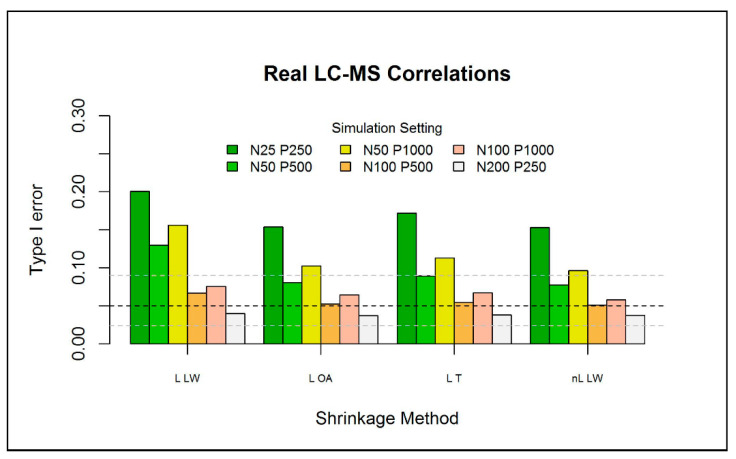
Type I error (over 200 simulations) for the eigenvalue-shrinkage approaches for the MD (with $\chi^{2}$—based critical limits) for a P-variate normal distribution with 0 mean and type (iii) covariance matrix. The horizontal black dashed line indicates the expected type I error (0.05) with the respective binomial Clopper–Pearson interval (horizontal grey-dashed line), with parameters 0.05∗200 and 200. The test data has the same multivariate normal structure of the train data, with a fixed sample size of N=100, where P equals the size of the train data.

The type I error of the one-class models was further investigated for different numbers of reference observations N∈{25, 50, 100, 200}, metabolites P∈{250, 500, 1000}, and three correlation structures between the metabolites. Here, the results are presented for a correlation structure that was based on a real metabolomics dataset (i.e., type (iii)). The other results are presented in Section S6 of the Supplementary Materials. For each setting, 200 independent datasets are generated. For each simulation, we generate an additional 100 independent observations to be used as test data. As we focus on the type I error, we do not assume any abnormality in the test data. Figure 4 depicts the results of the different simulation settings for one-class models based on each of the 4 selected eigenvalue-shrinkage estimators. As expected, increasing the sample size, while keeping P fixed, largely improves the type I error for all methods (see also Figure S7 of the Supplementary Materials). Conversely, when the number of metabolites is increased, with N fixed, the type I error deteriorates. Overall, very low sample sizes (N=25) increase the type I error slightly over the nominal level and should therefore not be overlooked in similar practical applications. However, we will see in the next section that, in comparison to current (PCA-based) one-class models, the type I error is much closer to the nominal level. Generally, all eigenvalue-shrinkage estimators improve upon “L LW”, which was used by Ullah et al. [8] to construct their “ES-CM”, in the sense that their type I errors are much closer to the nominal level. 

Overall, the type I error of the one-class model based on the “nL LW” estimator was closest to the nominal level. However, the computationally much less expensive procedures “L OA” and “L T” showed comparable performance, with most type I errors well within the binomial Clopper–Pearson interval. In all simulations, one-class modeling in combination with “L T” eigenvalue shrinkage emerged as the fastest procedure with acceptable control over the type I error. L OA was a close second, but the “L T” approach is assumed to be a bit more robust to departures from normality, as explained in Section 4.5, since it is a non-parametric method. Therefore, ES-CM uses this eigenvalue-shrinkage approach. 

#### 2.3.3. Improved Power of ES-CM over the Standard PCA-Based Decision Criteria

**Figure 5:**
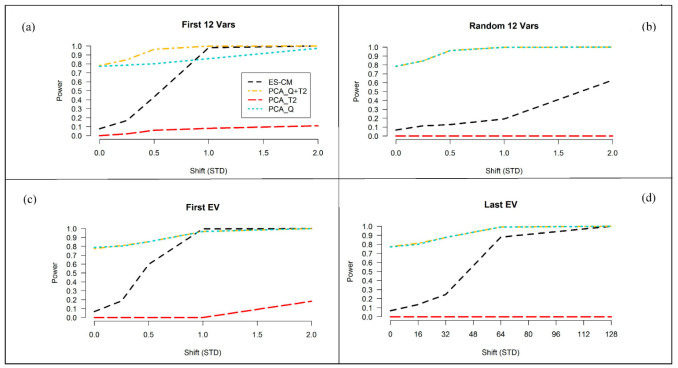
Percentage of correct detection of the introduced abnormality (over 200 simulations) for ES-CM based on “L T” shrinkage for the MD (with $\chi^{2}$—based critical limits) and for the standard PCA-based decision criteria (simulation 1). On the x-axis the magnitude of the shift (in standard deviations) in the mean between the reference population and the test sample and on the y-axis the percentage of correct detection of the introduced abnormality (i.e., power). The black line shows the detection percentage based on ES-CM, the red line the PC-based score distance alone, the cyan line the PC-based orthogonal distance only and the gold line the two PC-based measures combined. Each simulation setting consisted of N=25 units and a (P=250)-variate normal distribution with 0 mean and a type (iii) covariance matrix.In panel (**a**), the test data (N=100, P=250) has a mean shift for the first 12 variables of size (0, 0.25, 0.5, 1, 2). In panel (**b**), the test data has a mean shift in 12 random variables of size (0, 0.25, 0.5, 1, 2). In panel (**c**), shifts of size (0, 0.25, 0.5, 1, 2) were done along the first eigenvector. In panel (**d**), shifts of size (0, 16, 32, 64, 128) were done along the last eigenvector.

Next, the power of ES-CM for outlier detection was examined. We consider the settings N=50, P=250, and N=100, P=1000 for the train data. Figure 5 presents the results for the latter setting. The simulation results for N=25 and P=250 are shown in Figure S8 of the Supplementary Materials. The four panels in Figure 5 correspond to four different types of abnormal metabolite profiles of the test observations in comparison to the reference class, namely (a) a difference in the first 12 variables (metabolites), (b) a difference in a random subset of variables, (c) a difference along the first eigenvector of the population covariance matrix of the references (i.e., shifts along directions where the references show the most variation), and (d) a difference along the last eigenvector (i.e., shifts along directions where references show the least variation). In each of these cases, the size of the abnormality, labeled “shift (STD)” in Figure 5, is tuned so that ES-CM showed intermediate power over most of the values of the sizes considered. 

As expected, for each scenario the power to detect outliers increases with increasing effect sizes. Case (a) and (c) share similar trends in the observed power, because the abnormalities introduced in the first variables are most likely associated to the largest eigenvalues. Figure S9 of the Supplementary Materials clearly shows that the covariance matrix used in the simulations has a blocked structure. The first 12 variables belong to a block of highly correlated variables, which are potentially associated to direction of large variance. We expect high detection power when using shrinkage in these settings, because it corrects for the error (i.e., overestimation) along the eigenvectors expressing major variance, enhancing the mean differences among the abnormal and the other metabolites and thus facilitating the detection of abnormal metabolite patterns along these directions [25]. In the other cases (see panels (b) and (d)), power is expected to decrease [25], i.e., when shifts are randomly introduced among the variables or expressed along the last eigenvalues. Particularly, the results in panel (b) refers to situations when outlying values are present in a random subset of (possibly uncorrelated) variables. We can see that compared to panel (a) and (c), the shrinkage statistic has less power against this type of abnormalities, which should not be picked up by the method too easily. In panel (d), we can see that the power is decreased when an abnormality is expressed along the eigenvectors associated to the smallest eigenvalues (i.e., lowly correlated metabolites). This is because shrinkage decreases the weighting given to the small eigenvalues [25]. Nevertheless, this is not very much alarming, since these types of shifts are not expected to occur much in practice.

Figure 5 also compares the power of “ES-CM” to that of a PCA-based one-class model. Note that in the figure we can also evaluate the type I error of two methods when the effect size is 0. Generally, outlier detection in PCA-based one-class modeling is based on two statistics (distance measures), namely Hotelling’s $T^{2}$ and the so-called Q-statistic [26,27,28]. These measures can also be combined for outlier detection. This way a single statistic can be used for outlier detection. Figure 5 seems to suggest that the PCA statistics PCA_Q and PCA_T2+Q offer more power to detect outliers in comparison to ES-CM. This, however, should not be misunderstood as a positive result, since their type I error (incorrectly marking a reference observation as outlying) is not taken into account. Note that the type I error of the methods can be seen in Figure 5 for shifts (*x*-axis) of zero. The type I error of PCA_Q and PCA_T2+Q was around 80%, which is clearly much larger than that of ES-CM (and the nominal level of 5%). In contrast, the PCA_T2 test appears to be very conservative (too low type I error) and hardly has any power to detect outliers. 

## 3. Discussion

In this paper, we have proposed a one-class model for the analysis of high-dimensional (metabolomics) data called ES-CM. The method combines estimation of Mahalanobis distances with an eigenvalueshrinkage estimator of the covariance matrix. It was shown that ES-CM outperforms PCA-based outlier detection and the shrinkage-based one-class model proposed by Ullah et al. [8].

It was shown in simulations that the type I error of ES-CM was close to the nominal level for datasets with 50–100 reference observations and up to 1000 variables. Additional simulations (results not shown) suggest that with 50–100 reference observations the type I error tends to increase when more variables (i.e., 2000–5000) are considered. For instance, for P=2000 we observed a type I error of 0.16, which is still substantially lower than the standard PCA-based approaches. The type I error is expected to decrease when a larger number of observations are available. Finally, the simulations have shown that ES-CM has high power to detect outliers, especially when an observation has outlying values in set(s) of correlated variables. The power was lower when outlying values were introduced in a random set of (possibly) uncorrelated variables. This is attributed to masking of outlying-ness by the normal variation in the other variables [29]. This effect is assumed to be larger for datasets with more variables.

ES-CM employs a modern eigenvalue-shrinkage estimator of the covariance matrix of the reference class. As detailed in Section 4.5, this estimator can be seen as an eigenvalue-shrinkage approach, but also as a weighted average of the sample covariance matrix and a target matrix with a simple structure. Here, the identity matrix was chosen as the target, but other targets could be employed as well [15] As the data was auto-scaled, the use of the identity matrix as shrinkage target implies that in ES-CM the sample correlations are shrunk towards zero.

Alternatively, in ES-CM, one could aim to impose more specific structure on the (inverse of the) covariance matrix by assuming that many of the (partial) correlations among the metabolites are exactly 0. Arguably, the Graphical LASSO (GLASSO) [30,31,32] is the most well-known sparse estimator that imposes such a structure. Currently, however, the method is computationally too expensive for application in ES-CM. Therefore, the use of structured covariance estimators was not considered further. It would be interesting, however, to explore the use of other modern covariance estimators in a one-class modeling context, such as the methods reviewed by Kuismin et al. [31]. 

ES-CM was evaluated under the assumption that the distribution of the reference class is multivariate normal. In practice, when this assumption is not met, a Box-Cox transformation (like the logarithm used in case study 1 of the present paper, or the square root) can be applied to make the data more normal distribution-like. Note that the “L T” estimator of the covariance matrix used by ES-CM is quite robust against departures from normality assumptions [15]. An interesting direction for future research would be to evaluate the robustness of ES-CM itself as well. Initial investigations suggest that the methods are quite robust as well: for a few selected simulations (not shown), it was observed that the type I error of ES-CM was close to the nominal level when the distribution of the reference class was multivariate t with 1 degree of freedom instead of multivariate normal. 

Similar to many statistical approaches, outliers in the training data can affect the performance of ES-CM. Therefore, in case study 2, a clinical expert interpreted the 1H-NMR spectra to ensure that no obvious outliers were present. Additionally, in both case studies, cross-validation was used as a first practical solution to detect potentially outlying observations in the training data. For, this purpose, the stability of the critical value of ES-CM during cross-validation was investigated, as well as the cross-validated values of the eigenvalue-shrinkage MD. In case study 1, 2 potential outliers were identified this way, but the results provided in Section 2.1 remained largely the same when these were excluded from the analysis. Alternatively, to circumvent outlier detection in the training data, estimators of the mean and the covariance matrix that are robust to (some) outliers in the training data could be employed [33,34,35,36,37,38,39,40,41], but further research is needed to explore the use of such methods in combination with eigenvalue-shrinkage in high-dimensional data. 

We have demonstrated the ability of ES-CM using 2 high-dimensional metabolomics datasets. In case study 1, ES-CM was used to explore metabolite profiles of cisgenic potato varieties in light of normal crop variation. Cisgenic potato varieties were mostly not identified as outliers in the safety assessment of experimental potato tubers, which is in line with the practical expectations of the current research field [4]. It is also well known that the natural biochemical variation between plants of the same cultivar (i.e., commercial plant) can be large, as a result of the different growth environments and harvest practices. This explains the non-compliance to ES-CM of the potatoes from other geographical locations in our case study. Secondly, we have revised the study in Engel et al. [3] focusing on diagnosis of inborn errors of metabolism (amongst other abnormalities) with our ES-CM. Our approach focused on a MD-based one-class model, whereas the previous study used a PCA-based one-class model, exploiting the Q statistic as detection criterion. Comparing Figure 5 in the present paper with the results shown in Engel et al. [3], we can conclude that their one-class model has a slightly higher detection power. In fact, the use of the Q statistic only filters out the normal metabolic variation of the reference class and consequently geometrically operates in the space orthogonal to the principal component subspace. In this study, we can reasonably assume that the IEM diseases are orthogonal to the principal component subspace and thus easily detectable as abnormal. However, the popular PCA-based approaches always require the optimization of the number of PCs which determine the split into PC and residual subspace, and currently no clear protocol has been defined. Remarkably, the geometrical partition of the modelled data variability might also suffer from the smearing effect [5]. 

We believe that our new approach is certainly more general, whenever no prior knowledge is available on the test data (and their related type of abnormality). Secondly, ES-CM offers a more comprehensive diagnostic, covering the entire variables space, to detect strange abnormalities with respect to the normal metabolic expression of the reference set, as in the case of the IEM patients.

## 4. Materials and Methods

### 4.1. Case Study 1: Compositional Safety Assessment of Tubers from Commercial and Cisgenic Potato Varieties 

For this study, 44 commercial potato varieties from traditional breeding (41 consumption and 3 starch varieties) and 8 cisgenic potato varieties were grown in a net cage in Wageningen, the Netherlands, by Wageningen University & Research. Seed tubers were planted according to a randomized block design with six blocks. The GM plants selected for this case study were cisgenic potato varieties that were enriched with different late blight resistance genes from crossable species [21]. The main purpose of this experiment was a comparative assessment of the cisgenic varieties to a reference collection of traditionally bred potato varieties. For 10 of the 41 non-starch varieties, tubers obtained from 2 other locations were analyzed as well. See Table S10 in the Supplementary Materials for a complete overview of the samples included in this study. After the vines were completely matured, the tubers were harvested in the first week of October 2018, collected from individual pots and stored at 4 °C until January 2019. For each variety and location, a pooled sample of randomly selected potato tubers from each of the 6 blocks plants was homogenized and freeze dried as described in Kok et al. [4], before their analysis by LC-MS. Samples (25 mg dry powder) were extracted in 1 mL of 75% methanol acidified with 0.1% formic acid according to De Vos et al. [42]. Chromatographic separation was performed on a HPLC system (Waters Acquity, Milford, MA, USA) with a C18-RP column (150×2.1 mm; Luna, Phenomenex) using a 5–35% acetonitrile gradient with 0.1% formic acid. Detection was done using an LTQ-Orbitrap FTMS hybrid mass spectrometer (Thermo Scientific, Bremen, Germany) in positive electrospray ionization mode. A mass resolution of 70,000 FWHM at a mass range of *m*/*z* 90–1350 was employed for data acquisition. Unbiased mass peak picking and alignment of the raw LC-MS data were performed using the MetAlign software [43]. From the resulting mass peak table of 81,120 mass features, signals present in <3 observations were filtered out and non-detects (i.e., peak intensity <1000 ions per scan) were replaced by a 0, using an in-house script called METalign Output Transformer (METOT). The remaining 18,214 individual mass peaks were subsequently clustered into so-called reconstructed metabolites (centrotypes), based on the correlation of mass peaks, derived from the same compound, in both their retention time and relative abundance across samples, using the MSCLust software [44]. The resulting metabolite dataset contains 1018 compounds (see Supplementary Materials), each represented by their in-source mass spectra, consisting of the (putative) molecular ion, natural isotopes, adducts, and fragments generated in the ion source. The dataset was further processed in the software R, version 4.0.4. First, metabolites with more than 40% (across the 61 samples) of 0’s (non-detects) were discarded, leaving 458 metabolites. The remaining 0 values in the dataset were imputed using random forest [45], and finally, the dataset was log-transformed and auto-scaled (0 mean and unit variance). Note that similar results as described in Section 2.1 were observed if missing values were imputed by half the minimum value in the dataset, instead of using random forest. 

### 4.2. Case Study 2: Diagnosis of Inborn Error of Metabolism

Data from a previously published study [3] are analyzed. Briefly, urine metabolite profiles were acquired from by 1H-NMR using a Bruker 500 MHz spectrometer from 218 Dutch (in the Gelderland region around the city of Nijmegen) subjects, aged between 4–12 years (with same proportion between males and females). The study group involved a set of 176 healthy children, 17 additional healthy children with exogenous metabolites related to diet, 18 patients diagnosed with IEM and 6 additional patients with particular drug intake. The regions 0.2–4.7 ppm and 5.0–10.0 ppm in the NMR spectra were selected for further analysis. The urine profiles were normalized to the creatinine signal to correct for dilution effects. Next, equidistant binning with a bin size of 0.04 ppm was used to reduce the dimension of the normalized data from 30,888 to 246 variables (bins) [3]. Finally, the data was centered to 0 mean and scaled to unit variance.

### 4.3. Simulation Set Up

In each simulation, we draw N reference observations (training data) from a P-variate normal distribution with mean μ and covariance matrix Σ. Three structures for Σ are considered, which are further illustrated in Figure S9 in the Supplementary Materials: (i) Independent block covariance structure, with block size of 25 variables. The within-block correlation is set to 0.8 and all variances to 1. (ii) Correlated blocked covariance structure. Extending structure (i), we allow the outer block correlations to be uniformly distributed between 0 and 0.4, whereas the within blocks (of fixed size 25) correlations are selected from a uniform distribution [0.6, 0.9]. All variances are set to 1. (iii) Correlation structure based on a real LC-MS metabolomics data [46], where we have used the pairwise correlations between the P variables with the highest variance. The study associated to this data involved a very large number of observations, which allows a proper calculation of the sample covariance matrix and therefore it can be considered representative enough of the true population covariance matrix. 

Reference observations are simulated by drawing random values from a multivariate normal distribution with μ=0 and one of the covariance structures. To investigate type I errors of the one-class model, for each simulated training data set, 100 test observations were drawn from the same distribution. To investigate the power for outlier detection, for each simulated data set 100 test observations were drawn from a multivariate normal distribution with mean $\mu_{T}$ and covariance matrix Σ. The test mean was different from the reference mean in four ways, namely (a) with a perturbation γ in the first 12 (highly correlated) variables, (b) as in (a), but with a perturbation γ touching 12 randomly highly and lowly correlated metabolites, (c) with a perturbation along the eigenvector of the covariance matrix Σ along the direction of maximum variance. The size of the perturbation is $\gamma \sqrt{\lambda_{\max}}$, with $\lambda_{\max}$ corresponding to the associated largest eigenvalue of Σ and (d) as in (c) with a perturbation along the eigenvector of minimum variance (touching lowly correlated variables), i.e., $\gamma \sqrt{\lambda_{\min}}$. We varied the magnitude of the shift γ to ensure intermediate power over most of the values of the sizes considered. Each simulation is repeated 200 times, each with a new set of reference observations and testing objects. Based on the similarities and the consistencies of the results the 3 covariance structures for the study on the type I error, we have only shown the results on the type (iii) covariance structure in Section 2.3 of this paper. Section S11 of the Supplementary Materials contains the R code used for the simulation study. In this last simulation, ES-CM was compared to a popular PCA-based one-class model (SIMCA), implemented in R package “mdatools” [26]. For simplicity, we have retained a number of principal components explaining at least the 80% of the variance of the reference observation.

### 4.4. One-Class Modelling Based on MD

Let N×P matrix X denote the data matrix of a sample of reference observations, whose P variables (metabolites) follow a multivariate normal distribution (after suitable transformations) with mean μ and covariance matrix Σ. We have also assumed that the set of references contains no outliers. In the text below, it is assumed that all data (including new observations) is auto-scaled using the estimates of the mean and standard deviations of X. 

The squared Mahalanobis distance is used to measure the difference between the metabolite profile of a new observation $x_{n}$ and the center of the reference class. It is estimated as ${\mathrm{MD}}^{2}\left(x_{n}\;\right)=\left(x_{n}-\hat{\mu }\right){\hat{\Sigma }}^{-1}{\left(x_{n}\;-\hat{\mu }\right)}^{T}$, with $\hat{\mu }$ and $\hat{\Sigma }$ the estimates of the population mean μ and covariance matrix Σ of the reference class. Traditionally, $\hat{\mu }$ and $\hat{\Sigma }$ correspond to the sample estimators of the mean and covariance matrix (here denoted as S)**.** The observation $x_{n}$ is marked as an outlier (classified as outside the one-class) if ${\mathrm{MD}}^{2}\left(x_{n}\right)>{\mathrm{MD}}_{\mathrm{crit}}^{2}$, where ${\mathrm{MD}}_{\mathrm{crit}}^{2}$ is a critical value that easily follows from an F distribution with P and N−P degrees of freedom [28], given a predetermined confidence level α. Thus, the MD together with the critical limit defines the one-class.

The MD estimate strongly relies on the (inverse) of the sample estimate of the covariance matrix of the reference class. This might present challenges. Figure 1 (panel (a)) shows that, even when N>P, the eigenvalues of the sample (diagonal) covariance matrix are overdispersed and estimated with large variance. For a general covariance structure, this means that the large eigenvalues tend to be overestimated and the small eigenvalues underestimated. As P~N, this problem is worsened, as illustrated in the same figure, panel (b). The numerical inversion to calculate the precision matrix will only amplify the estimation error. When P>N, the matrix S is rank-deficient (not positive definite) and thus it cannot be inverted (singular). As a consequence, a growing number of eigenvalues are 0, as illustrated in Figure 1, panel (c). These issues can negatively affect the power of the MD statistic for N>P settings and completely prevent its application in high-dimensional settings (N≪P). Hence, one-class modeling based on MD is typically not possible in untargeted metabolomics. 

Furthermore, the size of the reference set may potentially affect the detection power of the one-class models. In fact, when the sample size is small, there is too little amount of observation to properly characterize the multivariate distribution among the variables of the train data. As a consequence, we expect a large difference among the covariance matrix of the train set and the one of the test objects. Table S12 of our Supplementary Materials illustrates this interesting behavior by comparing the sample moments of the MD for two simulated sets of reference and test sets (with the same distribution) for different N,P settings. Cross-validation has been proposed as a remedy in Ramaker et al. [13] to improve the characterization of the MD and consequently improve the reliability of the MD threshold, especially for P≫N and small N. Therefore, it has been adopted throughout this paper as a prior step before any further data elaboration. 

### 4.5. (Shrinkage) Estimators of the Covariance Matrix

Shrinkage approaches adjust (shrink) the eigenvalues of the sample covariance matrix in a data-dependent manner to correct for their overdispersion (bias). This way an improved estimator is obtained. It can be shown that this estimator always returns a positive definite covariance matrix. Hence, combining the MD with eigenvalue-shrinkage offers an interesting route to one-class modeling in untargeted metabolomics, even when N≪P. Figure 1 (panel 2(a)–2(c)) shows that eigenvalue-shrinkage indeed largely reduces the overdispersion of the sample eigenvalues. Figure S1a of the Supplementary Materials illustrates how this positively effects one-class modelling. Here, a shrunken estimate of the covariance matrix is compared to the sample and true covariance matrix. We can clearly see that initially the dispersion among the reference observations is overestimated in certain directions and underestimated in others. This is corrected by shrinkage, resulting in improved power for detecting outliers and improved type I error for correctly marking reference observations as non-outlying. 

The starting point of our investigations was the one-class model proposed by Ullah et al. [8]. This method used a linear eigenvalue-shrinkage estimator of the covariance matrix of the reference class. This shrinkage estimator (“L LW”) was proposed in 2004 by Ledoit and Wolf [14]. The shrinkage estimator is given by ${\hat{\Sigma }}_{\mathrm{LW}}=\left(1-\delta \right)T+\delta S$, where δ is the shrinkage intensity, usually determined by cross-validation [47], but analytical expressions for δ do exist as well for given specific structures of the target matrix [14,48,49,50,51]. Here, the identity matrix was used as shrinkage target, which is a typical choice considering that all data is auto-scaled. In principle, other targets could be used as well. For the identity target an analytical expression can be used to determine the optimal value for δ. This optimal indicates how much the sample eigenvalues of Σ are shrunk towards 1 (the diagonal elements of the target matrix T). For example, δ =0 implies no contribution of S to ${\hat{\Sigma }}_{\mathrm{LW}}$, i.e., the variance between the reference observations is assumed to be equal to 1 in all directions in multivariate space. In contrast, δ =1 implies no shrinkage and only the sample eigenvalues are used. Intermediate values for δ reveal the simultaneous contribution of T and S to ${\hat{\Sigma }}_{\mathrm{LW}}$. The “L LW” estimator is implemented in the R packages “corpcor” (or equivalently in “nlshrink” or “rrcov”) and all use the original choice of the penalty term proposed by Ledoit et al. 

In recent years, several improvements to “L LW” estimator were proposed. In the present paper it was investigated whether these also offer an improvement in the context of one-class modeling. Chen et al. [16] proposed an improved method to find the optimal value for shrinkage intensity δ when the data is multivariate normal. The authors showed that for normally distributed data the value for δ that is found is close to the optimal value. Therefore, the method is referred to as an oracle approximating estimator, “L OA”. Note that when the data is not multivariate normal the “L LW” estimator offers a better estimate of the covariance matrix [16]. The “L OA” estimator is implemented in the R package “CovTools”. 

Touloumis [15] showed that in settings where the target matric equals the true covariance matrix (T=I) the value for δ used by “L LW” is biased downwards in extreme high-dimensional settings. An improved procedure for tuning δ was therefore proposed. This estimator, “L T”, has the desirable property of being non-parametric, thus it does not rely on the distributional assumptions on the reference class. Through simulations, Touloumis proved that “L T” outperforms “L LW” in scenarios which involved normally and non-normally distributed data, especially in cases of small sample sizes. The improvement seemed not to be relevant when Σ deviates slightly from the target matrix T, where the “L LW” approach could still perform sufficiently good. The “L T” estimator is contained in the R package “ShrinkCovMat”.

Linear eigenvalue-shrinkage is an approximation to a nonlinear problem. Recently, non-linear eigenvalue-shrinkage methods have been proposed by Ledoit et al. [24,52]. This method is denoted as “nL LW”. The non-linear shrinkage estimator does not employ a shrinkage target T or a shrinkage intensity δ. Instead, each eigenvalue is shrunk separately through the use of the sample spectral density and its Hilbert transform. The authors argued that this approach has substantial benefits when P/N is small and the population eigenvalues are dispersed. Similarly, linear shrinkage is expected to perform poorly when higher-order effects are present among the original variables. In the opposite case, that is when P/N is large, and/or the population eigenvalues are close to one another, linear shrinkage captures most of the potential improvement over the sample covariance matrix [18]. This estimator is implemented in the R package “nlshrink” and will be denoted as “nL LW”. 

In a parallel work, van Wieringen et al. [17] proposed a ridge estimator of the precision matrix ${\hat{\Sigma }}_{\mathrm{vW}}^{-1}={\left[{\left(\delta I+0.25{\left(S-\delta T\right)}^{2}\right)}^{0.5}+0.5\left(S-\delta T\right)\right]}^{-1}$. It can be shown that this is equivalent to nonlinear shrinkage of the sample eigenvalues. The R package “ragstoridges” contains the algorithm for estimating ${\hat{\Sigma }}_{\mathrm{vW}}^{-1}$, which will be denoted as “vW ridge”. Consistently with the authors, we have applied a cross-validation procedure for the choice of the optimal shrinkage intensity δ. 

### 4.6. One-Class Modelling Based on Shrunken MD

For one-class modeling in high-dimensional data we propose to combine eigenvalue-shrinkage (Section 4.5) with the MD (Section 4.4): for a test observation y, ${\mathrm{MD}}_{\mathrm{shrink}}^{2}\left(y\right)=\left(y\;-\hat{\mu }\right){\hat{\Sigma }}_{\mathrm{shrink}}^{-1}{\left(y\;-\hat{\mu }\right)}^{T},$ with ${\hat{\Sigma }}_{\mathrm{shrink}}$ being the shrinkage estimate of the covariance matrix (Σ) of the set of references. Of the 5 shrinkage estimators described in Section 4.5, the “L T” estimate for ${\hat{\Sigma }}_{\mathrm{shrink}}$ offers the best compromise between computational efficiency and type I error control (see Section 2.3) and is therefore used by ES-CM. New observations with ${\mathrm{MD}}_{\mathrm{shrink}}^{2}\left(.\;\right)>{\mathrm{MD}}_{\mathrm{crit}}^{*2}$ are marked as outliers. In contrast to Section 4.4, the value for ${\mathrm{MD}}_{\mathrm{crit}}^{*2}$ is not straightforward to specify. The statistical distribution of the ${\mathrm{MD}}_{\mathrm{shrink}}^{2}$ for the reference observations is unknown due to the shrinkage that is applied. Consequently, the classical F distribution is no longer applicable. Based on the results from Pomerantsev et al. [27] for PCA-based one-class modeling, we propose a scaled $\chi^{2}$ distribution for ${\mathrm{MD}}_{\mathrm{shrink}}^{2}$. The scaling factor and the degrees of freedom are obtained from cross-validation by the following algorithm:

(1) For each reference observation $x_{n},$ from 1 to N:
  - Leave the observation $x_{n}$ out and compute the (shrinkage) covariance estimate of the N−1 set of references using “L T”.
  - Compute the MD for the left-out observation, ${\mathrm{MD}}_{\mathrm{shrink}}^{2}\left(x_{n}\right).$
(2) Calculate the critical limit ${\mathrm{MD}}_{\mathrm{crit}}^{*2}$ for ES-CM using the quantile $g_{1}\chi_{\alpha }^{2}\left(g_{2}\right)$ of the scaled $\chi^{2}$ distribution for a predetermined significance level α (i.e., 5%), given the scale factor $g_{1}={\hat{s}}^{2}/\hat{m}$ and the degrees of freedom $g_{2}=2{\hat{m}}^{2}/{\hat{s}}^{2},$ with $\hat{m}$ and ${\hat{s}}^{2}$ being the (sample) mean and standard deviation of the (cross-validated) ${\mathrm{MD}}_{\mathrm{shrink}}^{2}$ values.

Alternatively, cross-validation in combination with a non-parametric bootstrap could be employed [8]. However, as shown in Figure S13 of Supplementary Materials, initial investigations suggest that the $\chi^{2}$ approach performs better.

## Figures and Tables

**Table 1:** Evaluation of the computational time and type I error for one simulated dataset with N=25 P=250 and a type (iii) covariance matrix for the different classes of eigenvalue-shrinkage methods, see Section 4. One-hundred test observations (with the same distribution as the set of references) are used to compute the type I error. A significance level of 0.05 was used.

	L LW	L OA	L T	nL LW	nL vW
Time [s]	1.19	1.00	0.79	189.00	490.00
Type I error	0.14	0.097	0.087	0.079	0.081

## Data Availability

Data for case study 1 is available as Supplementary Materials. No new data were created or analyzed for case study 2.

## Supplementary Materials

The following are available online at https://www.mdpi.com/article/10.3390/metabo11040237/s1, Figure S1: Schematic overview of a bivariate one-class model based on (a) the sample covariance matrix and (b) the eigenvalue-shrinkage covariance matrix, Figure S2a: PCA score plot of the potato metabolomics data, Figure S2b: DLOOCV for the train MD values for the potato data, Figure S3a: PCA score plot of the IEM metabolomics data, Figure S3b: DLOOCV for the train MD values for the IEM data, Figure S4: Variable Selection, Table S5: Abnormal compounds present in urine spectra from 2 outlying patients, Figure S6a: Type I error (over 200 simulations) for the eigenvalue-shrinkage approaches for the MD (with $\chi^{2}$—based critical limits) for a a P-variate normal distribution with 0 mean and type (i) covariance matrix, Figure S6b: Type I error (over 200 simulations) for the eigenvalue-shrinkage approaches for the MD (with $\chi^{2}$—based critical limits) for a a P-variate normal distribution with 0 mean and type (ii) covariance matrix, Figure S7: Type I error (over 200 simulations) for the “L T” eigenvalue-shrinkage approaches for the MD (with $\chi^{2}$—based critical limits) for a a P-variate normal distribution with 0 mean and type (iii) covariance matrix (P=250), Figure S8: Percentage of correct detection of the introduced abnormality (over 200 simulations) for ES-CM based on “L T” shrinkage for the MD (with $\chi^{2}$—based critical limits) and for the standard PCA-based decision criteria (simulation 2), Figure S9: Heatmaps of the population correlation matrices, Table S10: overview of the potato samples analyzed in case study 2, Figure S11: R script example, Table S12: MD sample statistics (over 1000 simulations) based on “LW” shrinkage as a function of the sample size of the reference set, Figure S13: Type I error (over 200 simulations) for the “L W” eigenvalue shrinkage approaches for the MD with $\chi^{2}$- or bootstrap-based critical limits for a P-variate normal distribution with 0 mean and type (i) covariance matrix (P=250).
