# Supplementary material for: Improved One-Class Modeling of High-Dimensional Metabolomics Data via Eigenvalue-Shrinkage
Source: Metabolites. 2021 Apr 13;11(4):237. doi: 10.3390/metabo11040237 (PMC8069634; doi:10.3390/metabo11040237)
Supplement: Supplementary file 1 [file metabolites-11-00237-s001.zip › metabolites-1165785-supple-for conversion/metabolites-1165785-supplementary.docx]

Article

Supplementary Material:

Improved One-Class Modelling of High-Dimensional Metabolomics Data via Eigenvalue-Shrinkage

Alberto Brini,^1^* Vahe Avagyan,^2^ Ric C.H. de Vos,^3^ Jack H. Vossen,^4^ Edwin van den Heuvel,^1^ Jasper Engel.^2^

^1^ Eindhoven University of Technology, Department of Mathematics and Computer Science, 5600 MB Eindhoven, The Netherlands

^2^ Biometris, Wageningen University and Research, Droevendaalsesteeg 1, 6708 PB Wageningen, the Netherlands;

^3^ Bioscience, Wageningen University and Research, Droevendaalsesteeg 1, 6700 AA Wageningen, the Netherlands

^4^ Plant Breeding, Wageningen University and Research, Droevendaalsesteeg 1, 6700 AJ Wageningen, the Netherlands

***** Correspondence: a.brini@tue.nl

Received: date; Accepted: date; Published: date

1.

(b)

(a)

\


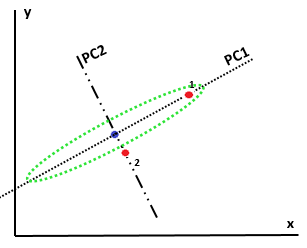

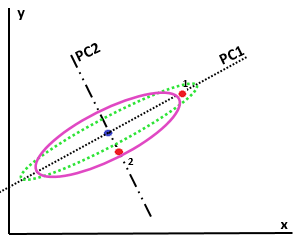


**Figure S1**. Schematic overview of a bivariate one-class model based on (a) the sample covariance matrix and (b) the eigenvalue shrinkage covariance matrix. The axes of the ellipses correspond to principal components PC1 and PC2. Panel (a) illustrates the class boundary of the one-class model based on the sample covariance matrix (green square dot ellipse), together with its centroid (blue dot) and two tests (red points). One test (red point 1) is along the direction of maximal variance of the reference data (PC1), and the second test (red point 2) is along the direction of minimal variance (PC2). The sample covariance results in the over- and underestimation of the variance in the multivariate space, as shown also in Warton et al.[1] A one-class model based on eigenvalue shrinkage pulls the highest sample eigenvalue downwards and the lowest one upwards, thus reducing the eccentricity of the elliptical boundary (solid purple). As a consequence, point 1 is marked as an outlier and point 2 is not.

2.


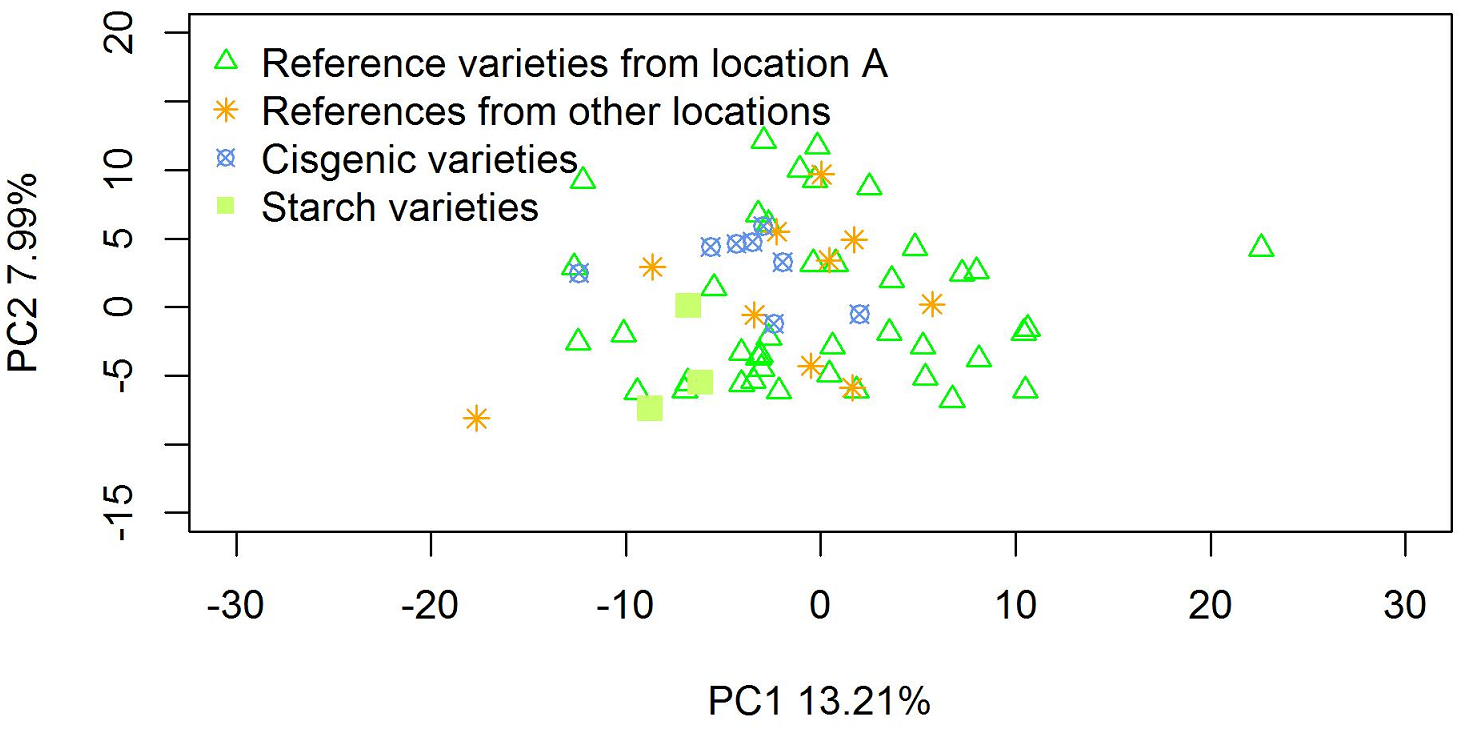


**Figure S2a**. PCA score plot of the potato metabolomics data. The potatoes in the test set (reference from other locations, GMs and starchy potatoes) were projected in the PCA subspace described by the reference group.


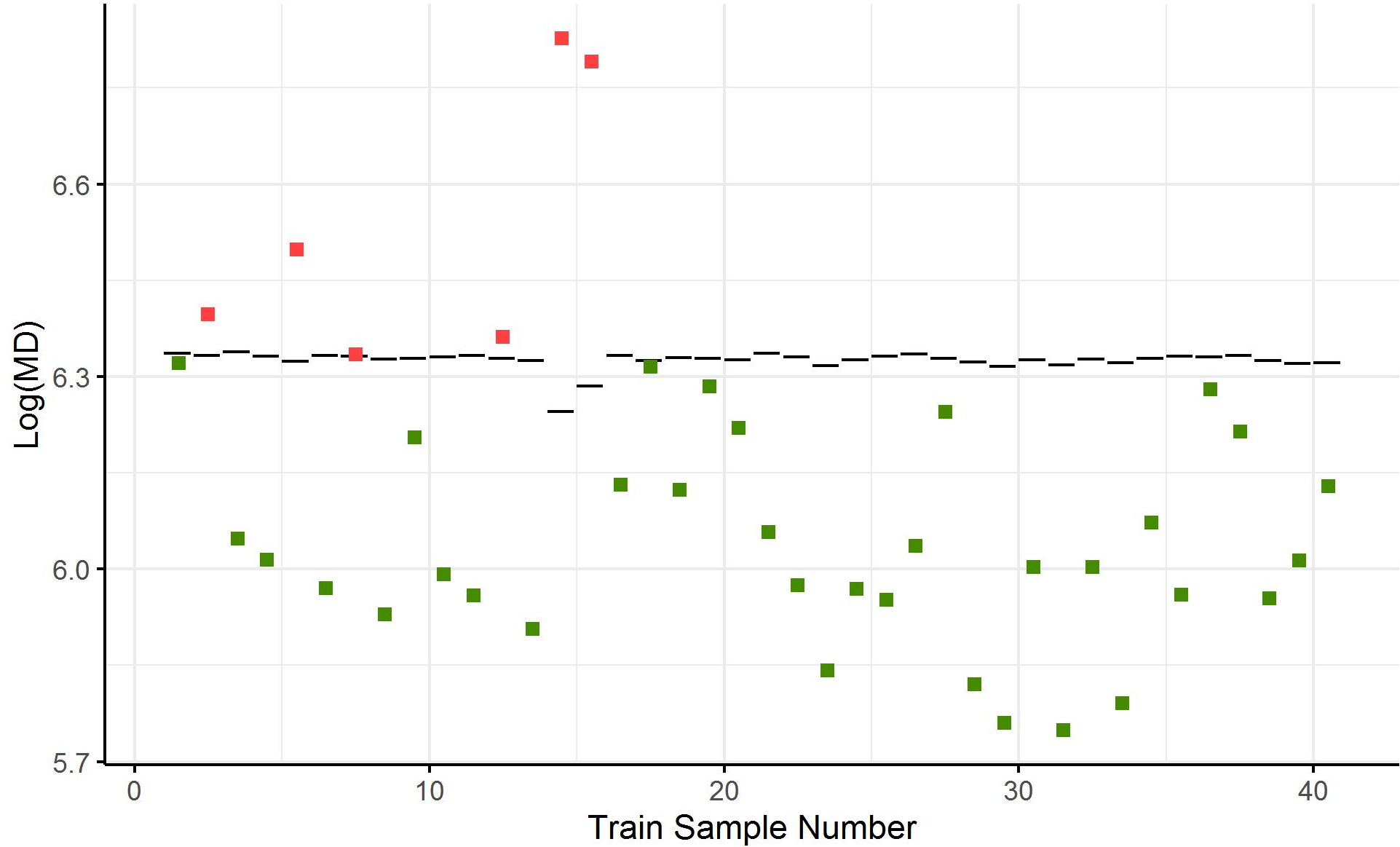


**Figure S2b**. DLOOCV for the train $\mathrm{MD}$ values for the potato data. Black lines represent the calculated critical limit. Squares are the calculated $\mathrm{MD}$ for the left-out training sample. Green color indicates a $\mathrm{MD}$ value smaller than the critical limit, contrarily the red color is used when the left-out sample has a $\mathrm{MD}$ value higher than the critical limit (black line).

***3.***

Figure S3a. PCA score plot of the IEM metabolomics data (colored according to a clinical expert as in Engel et al*.)*


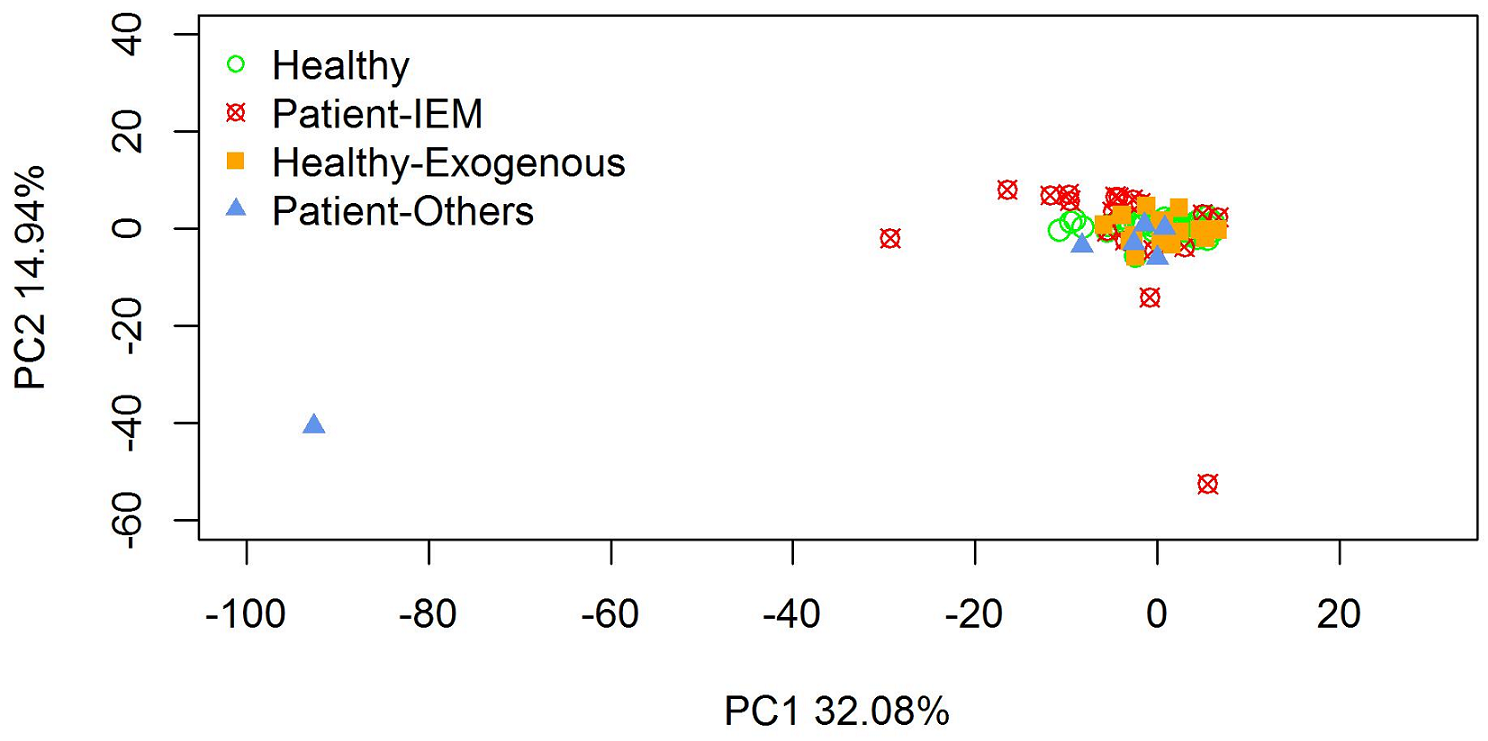


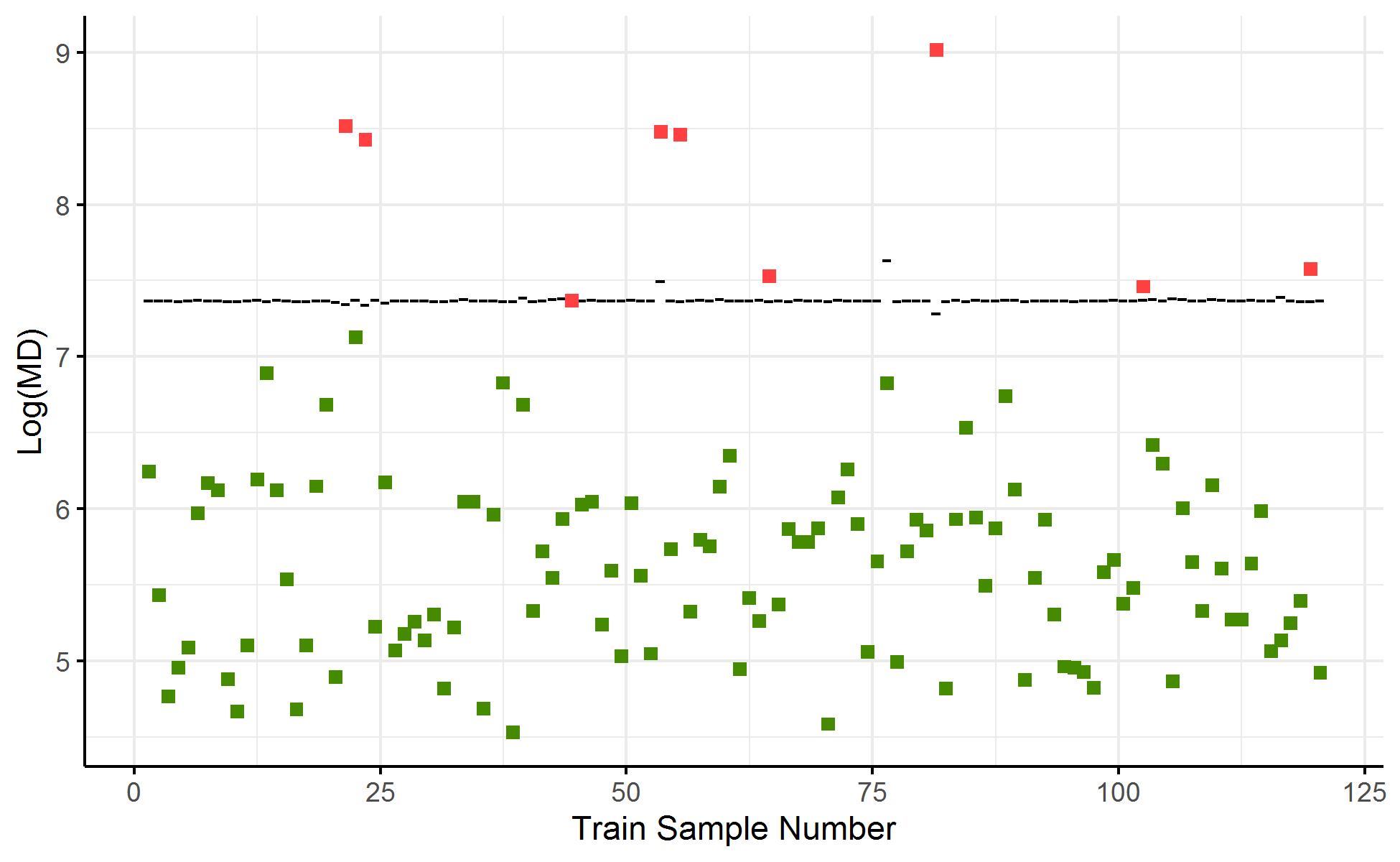


**Figure S3b.** DLOOCV for the train $\mathrm{MD}$ values for the IEM data (based on 120 healthy children). Black lines represent the calculated critical limit. Squares are the calculated $\mathrm{MD}$ for the left-out training sample. Green color indicates a $\mathrm{MD}$ value smaller than the critical limit, contrarily the red color is used when the left-out sample has a $\mathrm{MD}$ value higher than the critical limit (black line).

**4. Variable Selection**

In Whitening, the identification of the abnormal variables is performed by calculating the quantity $\mathbf{w=}(\mathbf{x}-\boldsymbol{\mu})\hat{Ʃ}^{-1/2}$ which quantifies the level of abnormality for each variable. The level values in $\boldsymbol{w}$ can be ranked based on their absolute value to identify the most abnormal variables. For the variable selection algorithm, we consider the LASSO (least absolute shrinkage and selection operator) norm penalized minimization problem

$$\min_{\boldsymbol{\beta}} \frac{1}{2}\left\| \boldsymbol{\beta}\hat{Ʃ}^{-1/2}-(\mathbf{x}-\boldsymbol{\mu})\hat{Ʃ}^{-1/2} \right\|_{2}^{2}+\tau\left\| \boldsymbol{\beta} \right\|_{1},$$

where $\boldsymbol{\beta}$ is a 1 × P sparse vector containing the variable abnormalities, i.e. all non-zero values are abnormal variables; τ is the shrinkage or penalization parameter which controls the intensity of the penalization and determines the sparsity of the estimated $\boldsymbol{\beta}$, and $\left\| . \right\|_{1}$ and $\left\| . \right\|_{2}$ are the LASSO and the Frobenius norm, respectively. We solved this optimization problem using the “glmnet” R package. Here, instead of determining the abnormality index per variable, we determine which variables are abnormal, using the shrinkage parameter τ. In principle, this approach might lead to a different ranking (by order of selection) of the variables compared to the whitening approach discussed before.

**5. Identification of the abnormal compounds in the IEM data**

| **Table S5.** Abnormal compounds present in urine spectra from 2 outlying patients. In bold, the ppm values associated to homogentisic acid.**Patient ID** | **CS; ppm multiplicity** | | **IEM/origin** |
| --- | --- | --- | --- |
|  | **Whitening** | **LASSO** |  |
| 197 | **3.63** **6.76** 3.13 **6.72** 4.29 4.06 6.68 | **6.72** **6.76** 6.80 **3.63** 6.57 3.05 8.85 | alkaptonuria |
| 185 | 3.13 4.29 4.17 3.67 2.04 3.63 3.86 | **9.67** **9.78** **9.74** **9.6**3 **5.13** **9.70** **2.16** | paracetamol |

It is known that Alkaptonuria is caused by a deficiency of the enzyme homogentisic acid oxidase in tyrosine catabolism which results in high concentrations of homogentisic acid in the urine. To find out which metabolites are indeed responsible of the IEM disease we performed a comparison of the abnormal metabolites found by the whitening and the LASSO methods, ranked in descending order, with a database of NMR spectra of model compounds. It is known that the abnormal singlet at 3.64 ppm and the multiplet at 6.78 ppm indicated that the metabotype of an individual contains a large concentration of homogentisic acid[3]. Both the whitening and the LASSO prove to be appropriate approaches for this purpose, selecting these two metabolites as source of outlyingness for the patient. We repeated the same procedure for the patient who consumed paracetamol. Due to the large number of resonances involved, identification of paracetamol intake via SHM is considered more difficult compared to the previous case. In fact, the two procedures identified different peaks in the NMR spectra. LASSO indicated relevant resonances around 9.8 ppm, which are known to be abnormal for this type of medication, probably associated to NH-groups in the molecule. Moreover, the singlets of 2.15 ppm and 5.13 ppm indicate that the metabolites acetaminophen, acetaminophen-glucuronide and acetaminophen-sulphate are present in high concentrations. The whitening approach selected instead a region of the spectrum (1.5-4.5 ppm) where abnormal peaks are detected, but not yet ascribed to well-known compounds.

**6.**


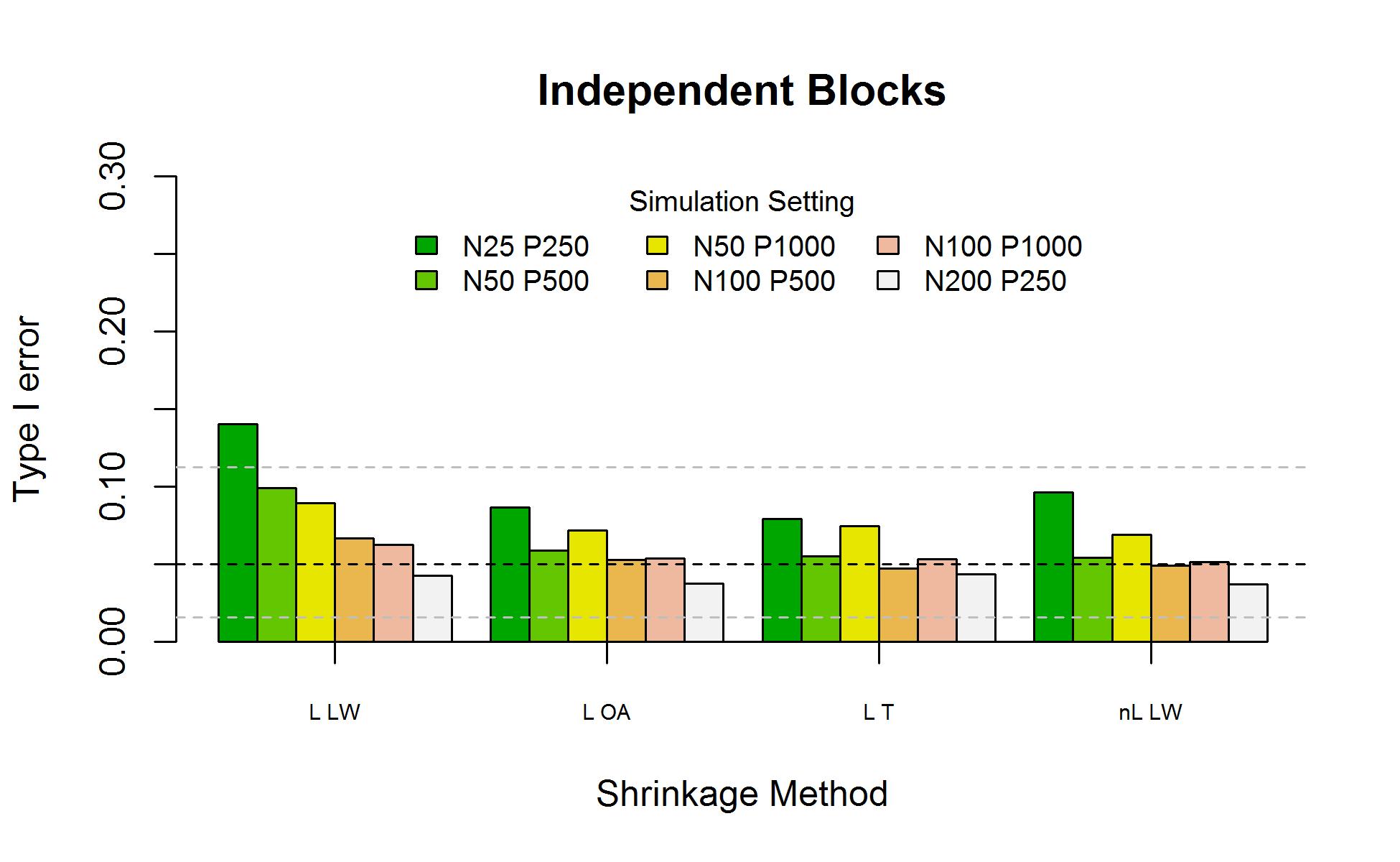


**Figure S6a**. Type I error (over $200$ simulations) for the eigenvalue shrinkage approaches for the $\mathrm{MD}$ (with $\chi^{2}-$based critical limits) for a P-variate normal distribution with $\mathbf{0}$ mean and type (i) covariance matrix. The horizontal black dashed line indicates the expected type I error ($0.05$) with the respective binomial Clopper-Pearson interval (horizontal grey-dashed line, with parameters $0.05*200$ and $200$. The test data has the same multivariate normal structure of the train data, with a fixed sample size of $N=100$, where $P$ equals the size of the train data.


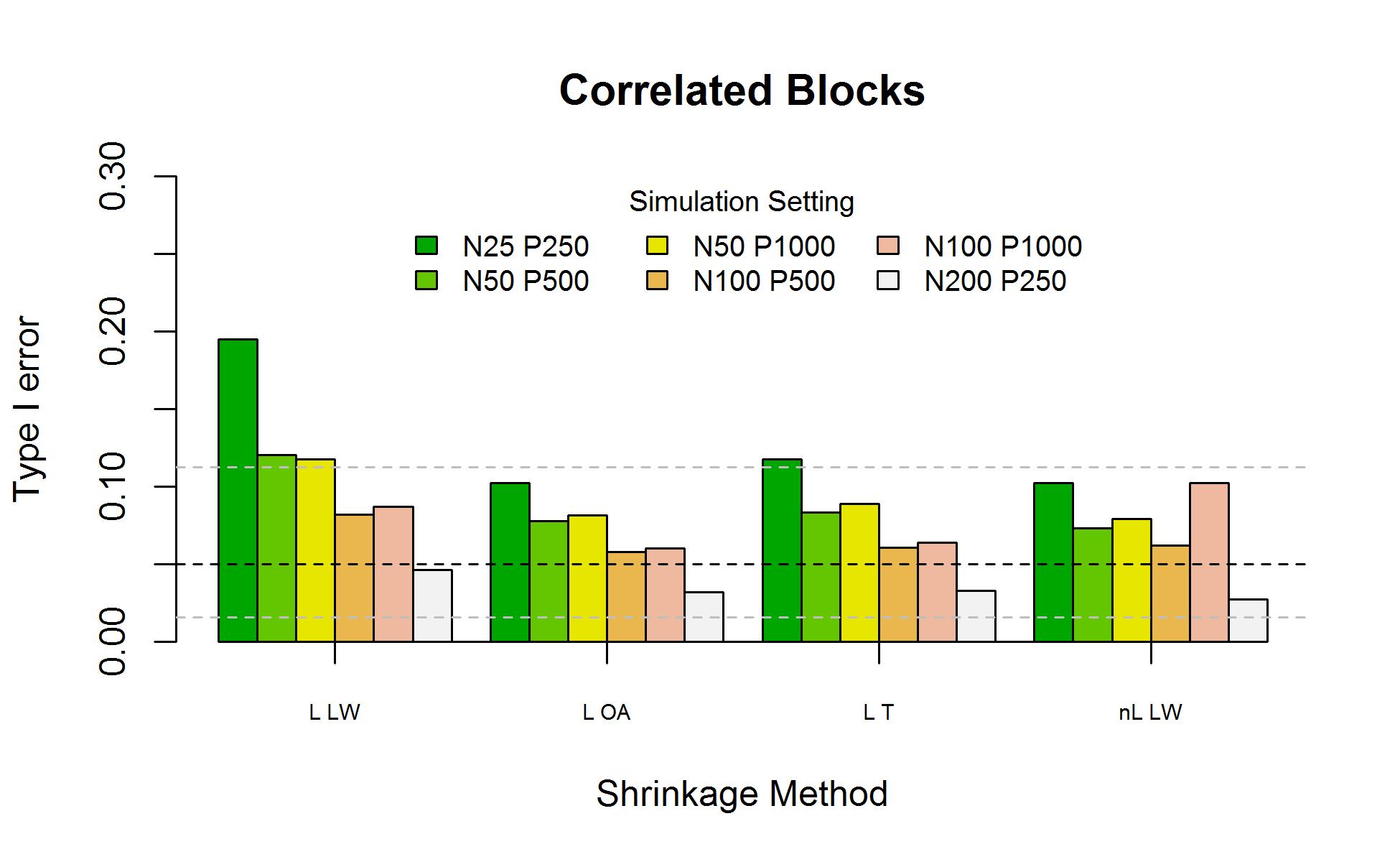


**Figure S6b**. Type I error (over $200$ simulations) for the eigenvalue shrinkage approaches for the $\mathrm{MD}$ (with $\chi^{2}-$based critical limits) for a a P-variate normal distribution with $\mathbf{0}$ mean and type (ii) covariance matrix. The horizontal black dashed line indicates the expected type I error (0.05) with the respective binomial Clopper-Pearson interval (horizontal grey-dashed line), with parameters $0.05*200$ and $200$. The test data has the same multivariate normal structure of the train data, with a fixed sample size of $N=100$, where $P$ equals the size of the train data.

**7.**

**Figure S7**. Type I error (over $200$ simulations) for the “L T” eigenvalue shrinkage approaches for the $\mathrm{MD}$ (with $\chi^{2}-$based critical limits) for a P-variate normal distribution with $\mathbf{0}$ mean and type (iii) covariance matrix ($P=250$). We have considered the samples size $N=25/50/100/200/250$. The horizontal grey line indicates the expected type I error ($0.05$) with the respective binomial Clopper-Pearson interval, indicated with horizontal grey-dashed line. The test data has the same multivariate normal structure of the train data, with a fixed sample size of $N=100$, where $P$ equals the size of the train data.


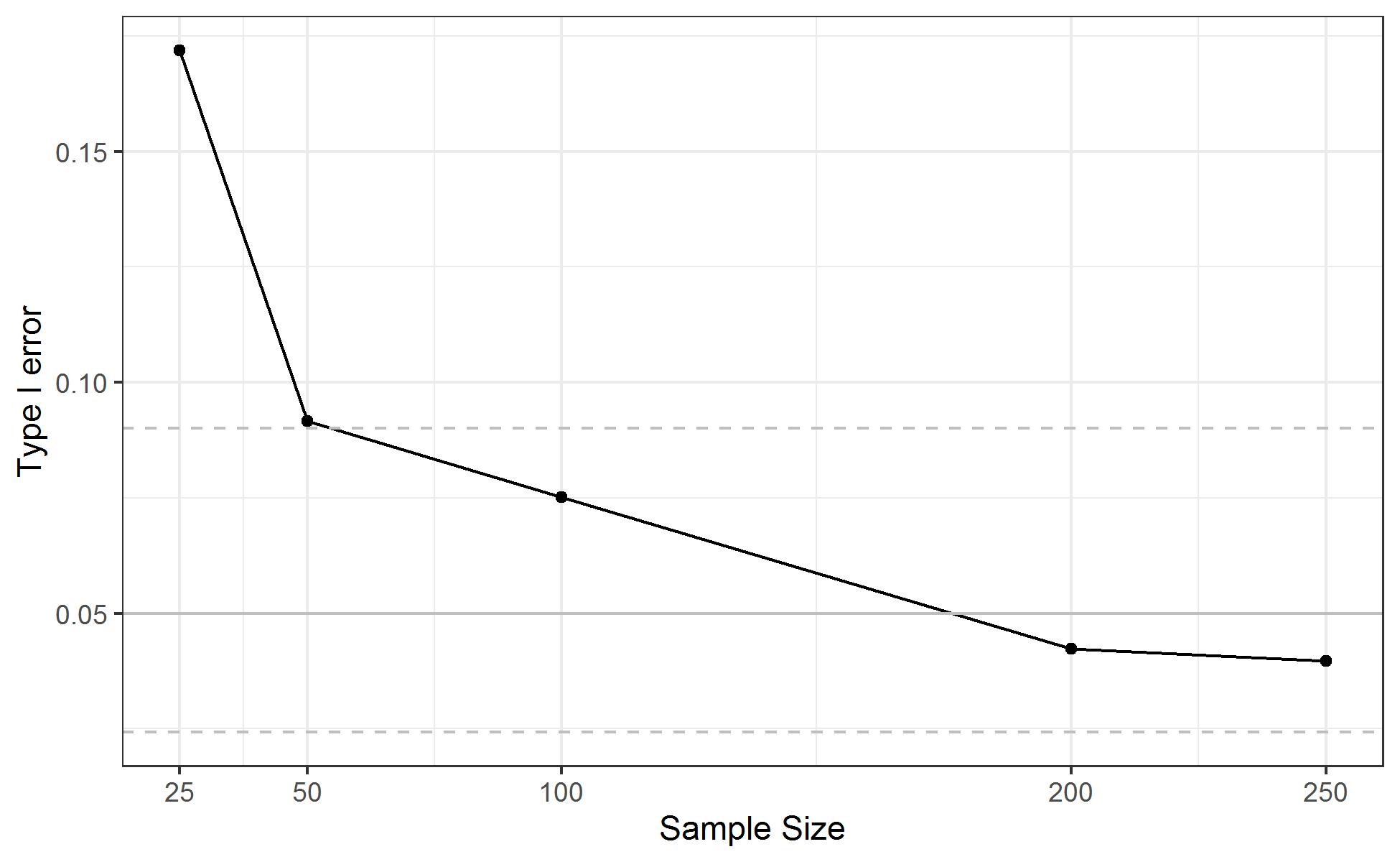

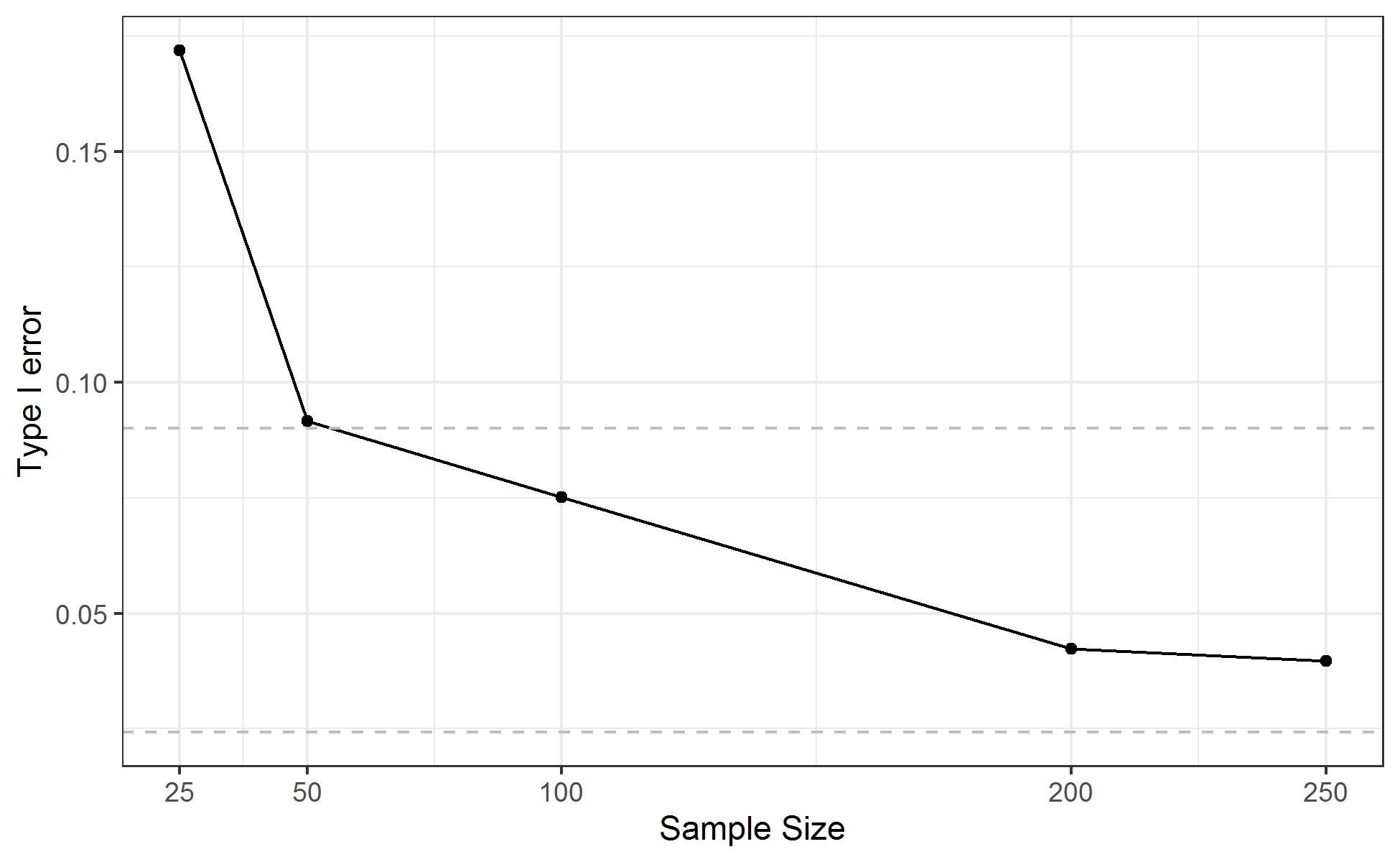

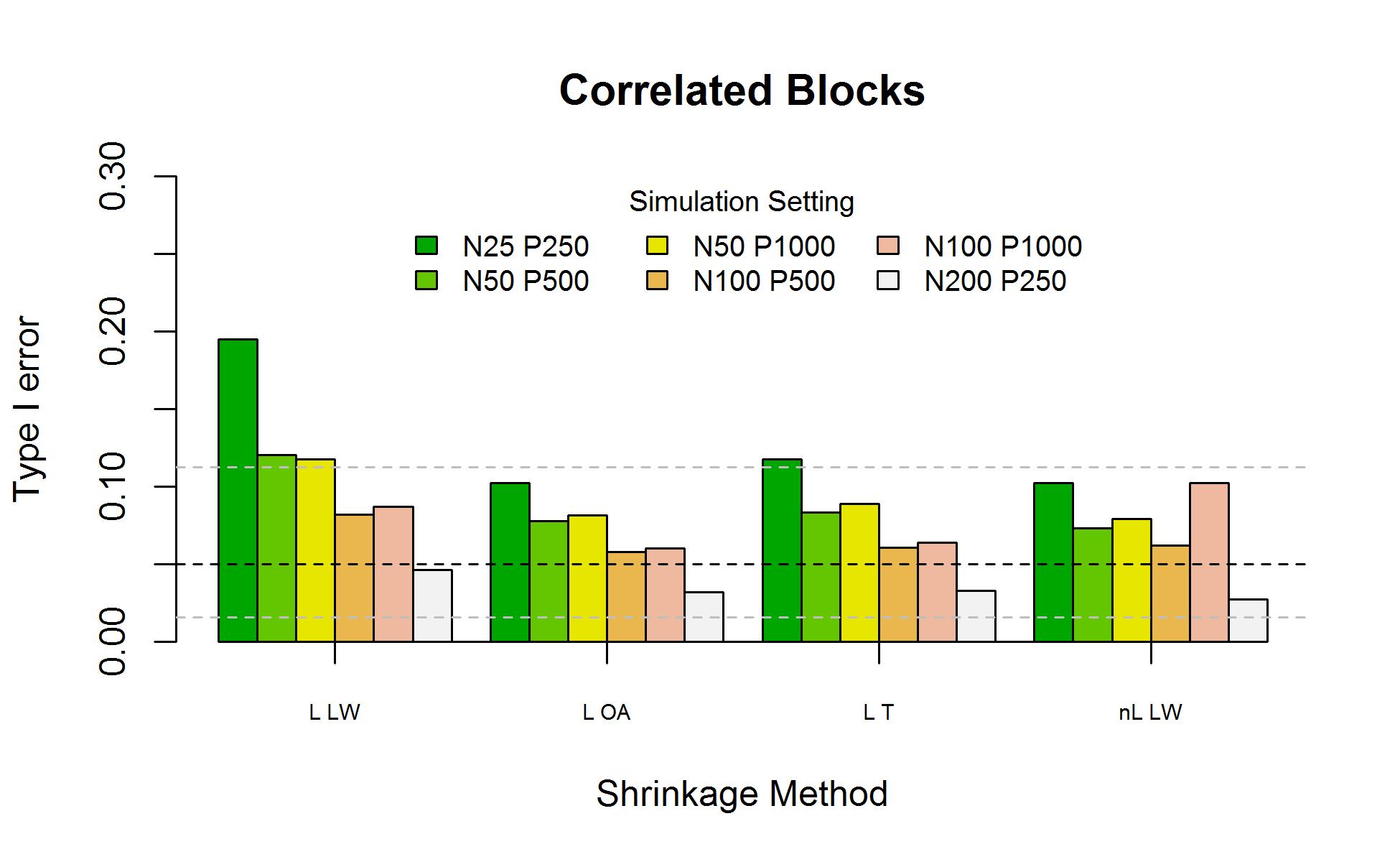


**8.** **Power analysis for** $\mathbf{N=25, P=250}$ **case.**

(c)

(b)

(a)

(d)


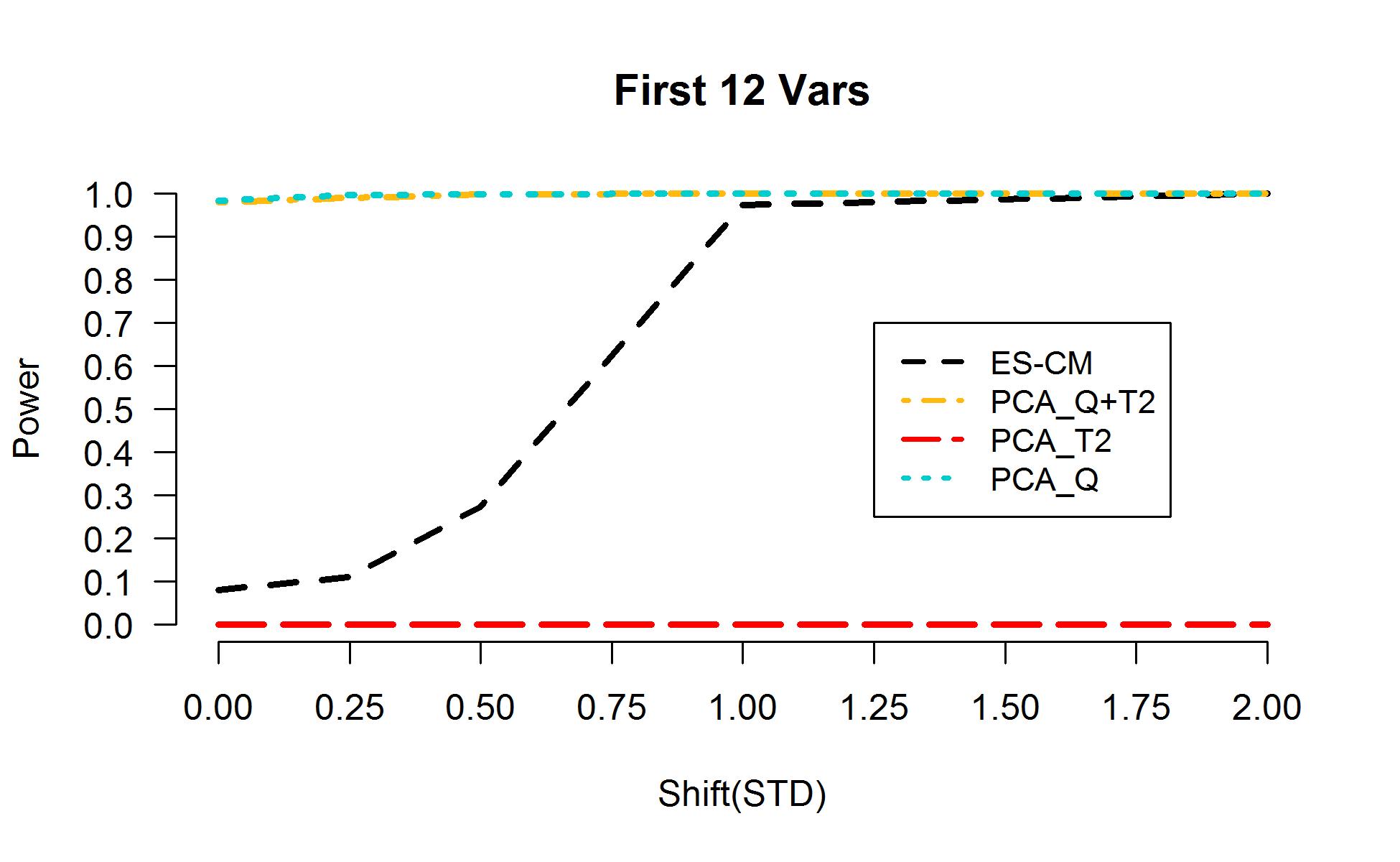

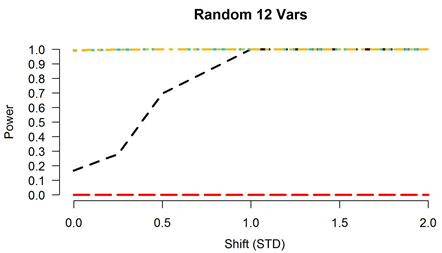

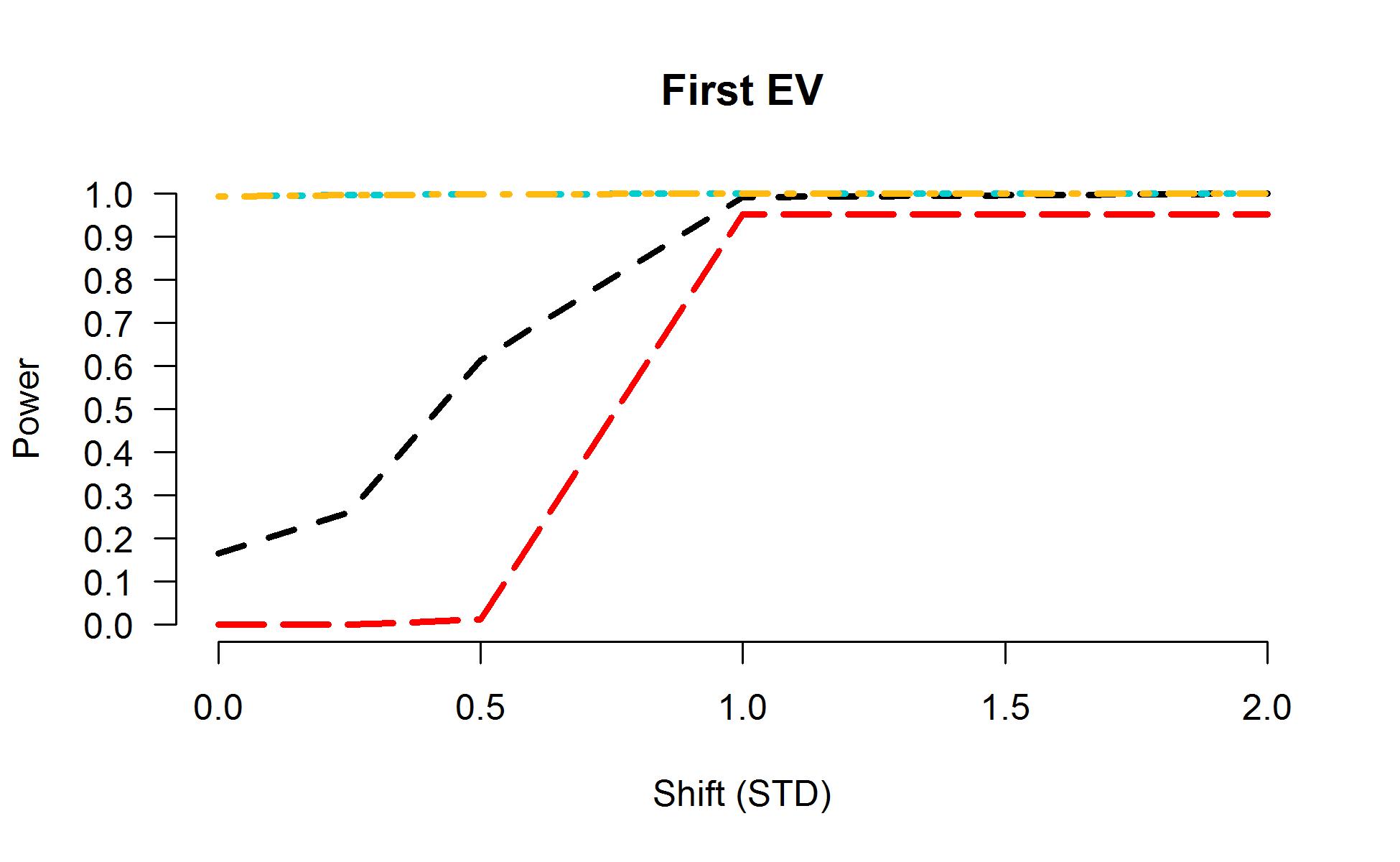

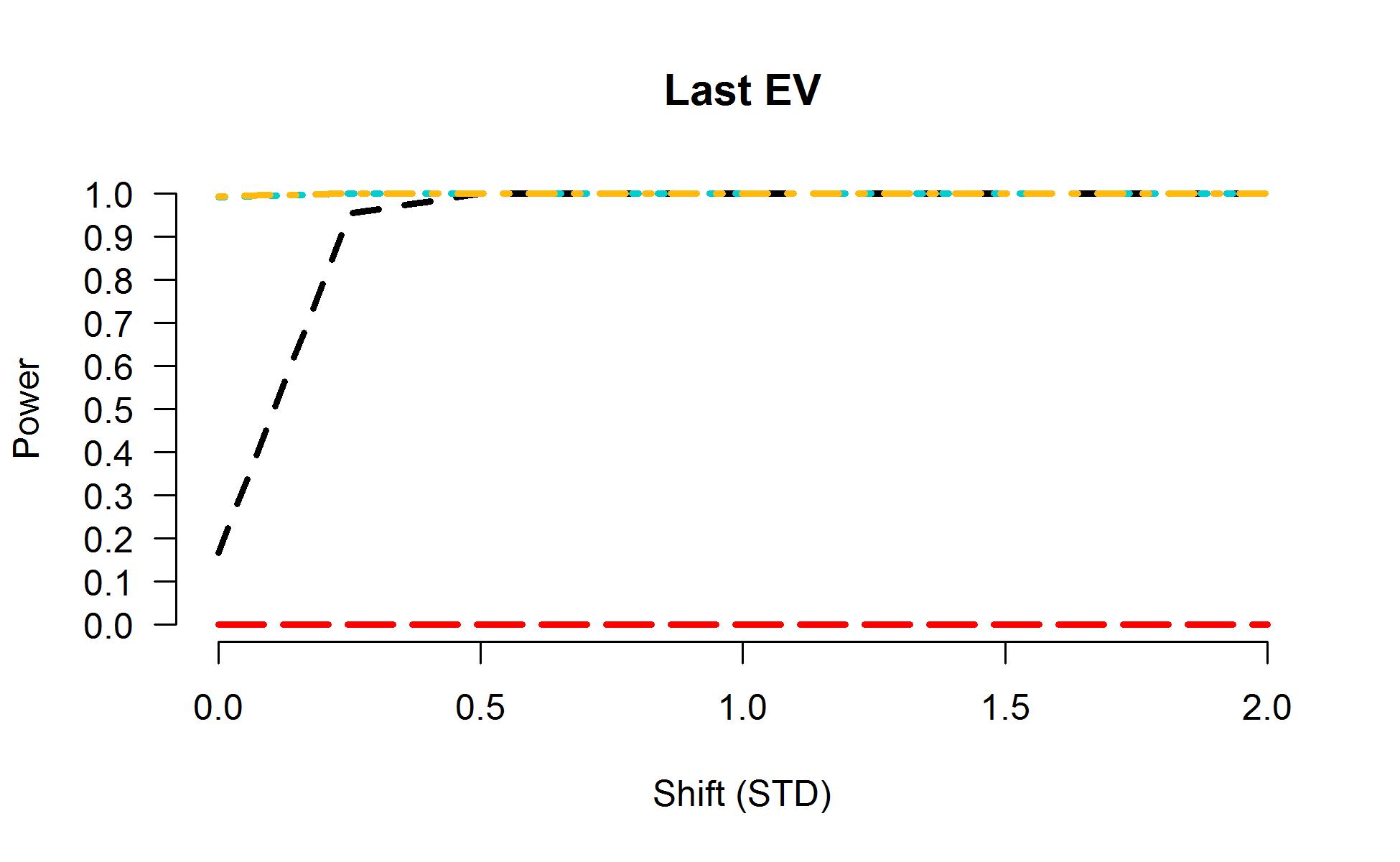

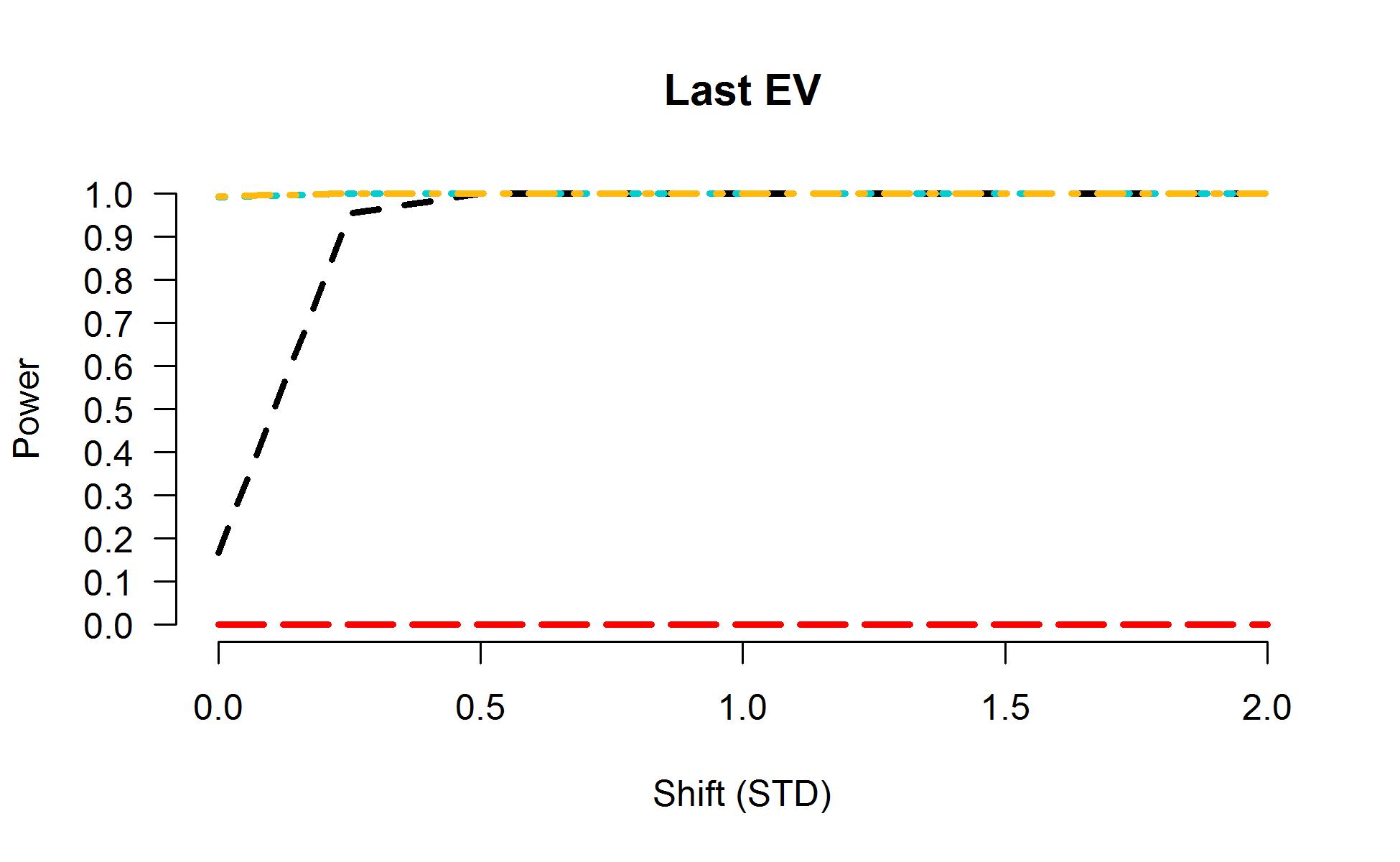


**Figure S8**. Percentage of correct detection of the introduced abnormality (over 200 simulations) for ES-CM based on “L T” shrinkage for the $\mathrm{MD}$ (with $\chi^{2}-$based critical limits) and for the standard PCA-based decision criteria (simulation 2). On the x-axis the magnitude of the shift (in standard deviations) in the mean between the reference population and the test sample and on the y-axis the percentage of correct detection of the introduced abnormality (i.e., power). The black line shows the detection percentage based on ES-CM, the red line the PC-based score distance alone, the cyan line the PC-based orthogonal distance only and the gold line the two PC-based measures combined. Each simulation setting consisted of training set of $N=25$ units and a ($P=250$)-variate normal distribution with $\mathbf{0}$ mean and a type (iii) covariance matrix. In panel (a) the test data ($N=100, P=250$) has a mean shift for the first $12$ variables of size ($0, 0.25, 0.5, 1, 2$). In panel (b) the test data has a mean shift in $12$ random variables of size $(0, 0.25, 0.5, 1, 2)$. In panel (c) shifts of size ($0, 0.25, 0.5, 1, 2$) were done along the first eigenvector. In panel (d) shifts of size ($0, 16, 32, 64, 128$) were done along the last eigenvector.

**9.**


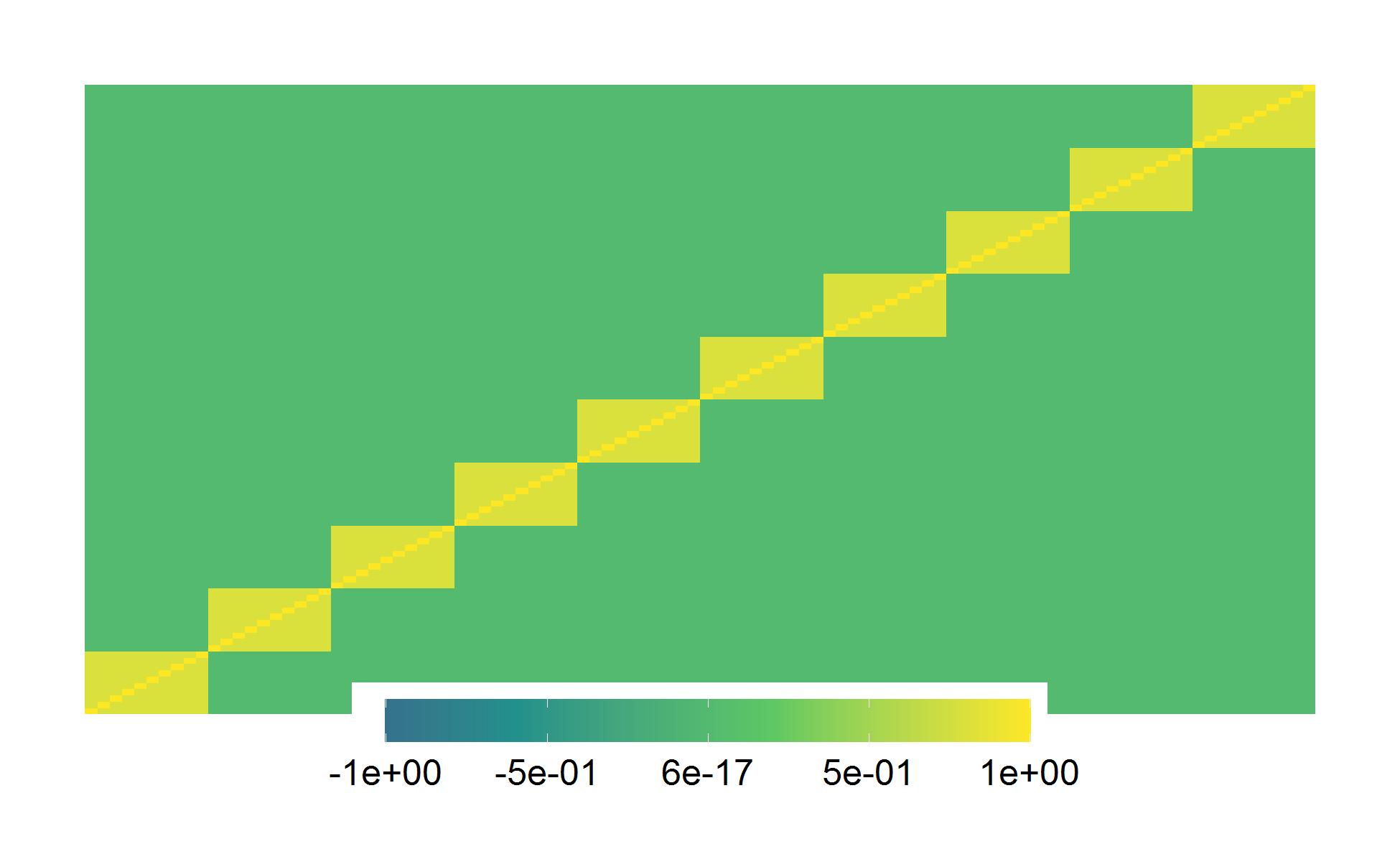

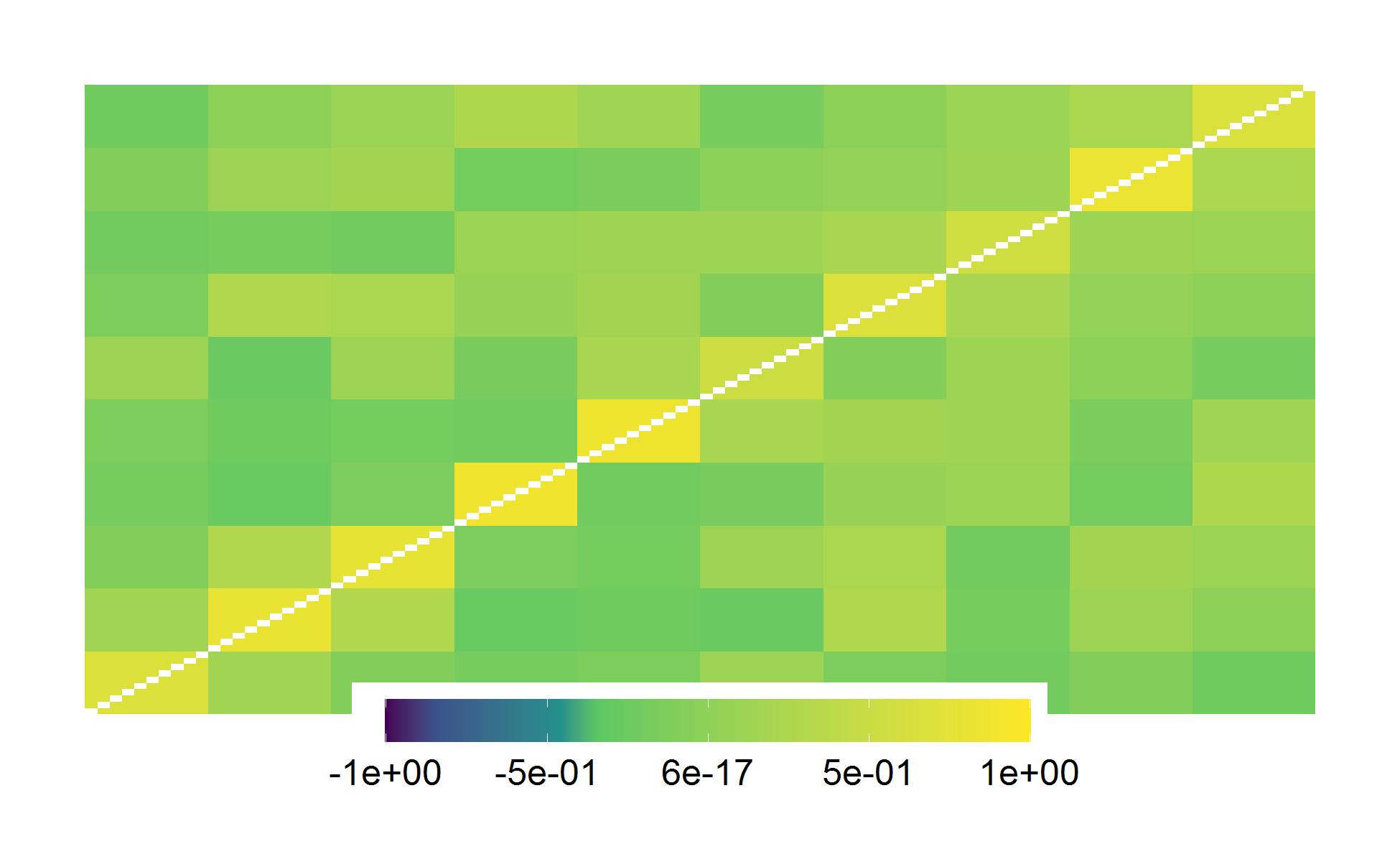


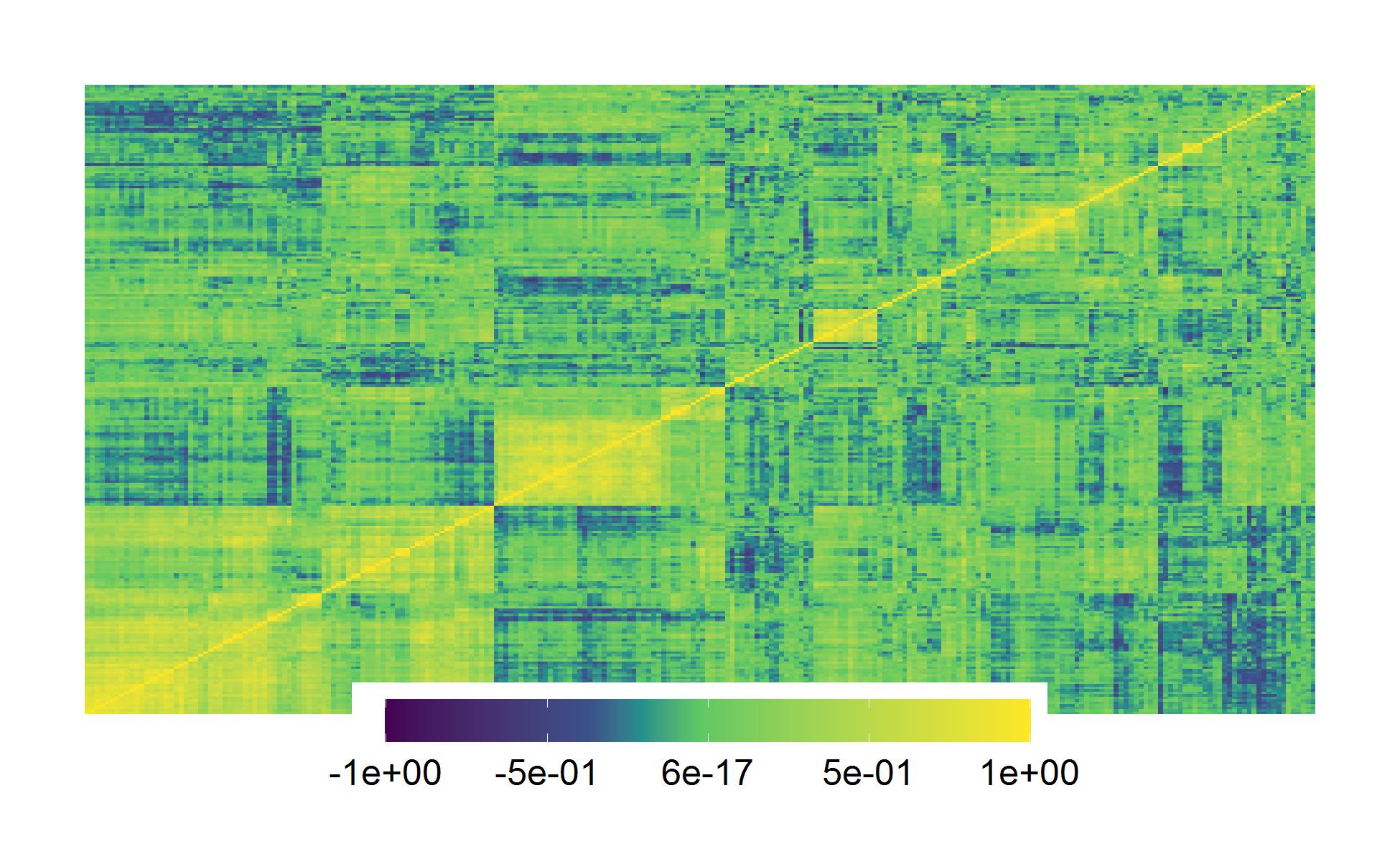


(iii)

(ii)

(i)

**Figure S9**: Heat maps of the population correlation matrices. . (i) Independent blocks (block size $25$ and within block correlations of $0.8$); (ii) correlated blocks (block size $25$ and within block correlations uniformly distributed [$0.6-0.9$] and outer block correlation uniformly distributed [$0.2; 0.4$]; (iii) a real LS-MS correlation matrix from the Husermet study [4] taking the $P$ variables with the highest variances. For the visualization, the P variables were clustered according to a hierarchical clustering algorithm (based on the average linkage method and the correlation matrix as similarity matrix)

**10.**

**Table S10**: Overview of the potato samples analyzed in case study 1. Note that the training data contains a set of varieties with a history of safe use for human consumption that were all sampled at the same location. Part of the test set contains these same varieties, but the potato material was obtained at two other locations. The test set also includes cisgenic events (the parent-commercial line is specified) and starch potatoes. These potato samples were obtained from the same location as the references in the training data.

| CULTIVAR | LOCATION | GROUP | DETAILS |
| --- | --- | --- | --- |
| PRESTO | A | Reference |  |
| smart | A | Reference |  |
| Karolin | A | Reference |  |
| GRAVITY | A | Reference |  |
| Deodara | A | Reference |  |
| Desiree | A | Reference |  |
| P | A | Reference | Parent P |
| Delcora | A | Reference |  |
| A | A | Reference | Parent A |
| Premiere | A | Reference |  |
| Mona Lisa | A | Reference |  |
| V | A | Reference | Parent V |
| CARNAVAL | A | Reference |  |
| VITALIA | A | Reference |  |
| LILY ROSE | A | Reference |  |
| Karlena | A | Reference |  |
| KIEBITZ | A | Reference |  |
| Celandine | A | Reference |  |
| Agata | A | Reference |  |
| Tomensa | A | Reference |  |
| Ultra | A | Reference |  |
| Compass | A | Reference |  |
| ANGELIQUE | A | Reference |  |
| Carrera | A | Reference |  |
| BIRTE | A | Reference |  |
| CHERIE | A | Reference |  |
| VR808 | A | Reference |  |
| Tivoli | A | Reference |  |
| B | A | Reference | Parent B |
| VALES EVEREST | A | Reference |  |
| FIDELIA | A | Reference |  |
| Kathadin | A | Reference |  |
| Talent | A | Reference |  |
| CUMBICA | A | Reference |  |
| Parella | A | Reference |  |
| SARPO MIRA | A | Reference |  |
| SASSY | A | Reference |  |
| Producent | A | Reference |  |
| ANTARTICA | A | Reference |  |
| MAYA | A | Reference |  |
| Annabelle | A | Reference |  |
| P110-48 | A | Test | Cisgenic (parent P) |
| P49-27 | A | Test | Cisgenic (parent P) |
| P110-16 | A | Test | Cisgenic (parent P) |
| P110-05 | A | Test | Cisgenic (parent P) |
| H43-4k | A | Test | Cisgenic (parent A) |
| F43-2 | A | Test | Cisgenic (parent: B) |
| V109-12 | A | Test | Cisgenic (parent V) |
| V109-17 | A | Test | Cisgenic (parent V) |
| Aveka | A | Test | Starch |
| Altus | B | Test | Starch |
| Altus | C | Test | Starch |
| smart | B | Test |  |
| Colomba | B | Test |  |
| P | B | Test |  |
| Carrera | B | Test |  |
| Desiree | B | Test |  |
| B | B | Test |  |
| V | B | Test |  |
| Parella | B | Test |  |
| Mona Lisa | B | Test |  |
| Colomba | C | Test |  |

**11. R script example .**

library(MASS)

library(ShrinkCovMat)library(mvtnorm)

simulate_data <- function {nref , p, rho, blocksize, ngmo, nabnormal, fsize, fault_type, var_idx) {…}

output <- simulate_data (nref = 150, p = 250, rho = 0.8, blocksize = 25, ngmo = 100, fsize = seq(4,4,by = 1), fault_type = 'specvar', var_idx = c(1:12), nabnormal = 12, cov r= R.data)

RawData <- output$ref

Test <- output$GMO

means <- colMeans(RawData)

scales <- apply( RawData,2,sd)

RawData <- sapply(1:ncol(RawData), FUN = function(col_idx){

(RawData[,col_idx] - means[col_idx])/scales[col_idx]})

Test <- sapply(1:ncol(Test), FUN = function(col_idx){

(Test[,col_idx] - means[col_idx])/scales[col_idx]})

nref <- nrow(RawData)

ntests <- nrow(Test)

precridge <- solve(shrinkcovmat.identity(t(RawData, centered=TRUE)[[1]])

MD_test_prec <- c()

for (T in 1:nref){

precridge.CV <- solve(shrinkcovmat.identity(t(RawData[-T,], centered=TRUE)[[1]])

MD_test_prec[T] <- rbind(RawData[T,])%*%precridge.CV%*%t(rbind(RawData[T,]))}

mean_Q <- mean(MD_test_prec)

var_Q <- var(MD_test_prec)o

Q_lim_cv <- (var_Q/(2*mean_Q))*qchisq(1-0.05, df = (2*mean_Q^2)/var_Q, ncp = 0, lower.tail = TRUE)

MD_test <- c()

for (n in 1:ntests) { MD_test [n] <- rbind(Test[n,])%*%precridge%*%t(rbind(Test[n,])) }

$$\boldsymbol{12.}$$

**Table S12**: $\mathrm{MD}$ sample statistics (over 1000 simulations) based on “LW” shrinkage as a function of the sample size of the reference set. Each simulation settings consisted a ($P=250$)-variate normal distribution with $\mathbf{0}$ mean and a type (iii) covariance matrix.. The test set is based on $100$ units with the same distribution as the reference data.

| **Sample Size / Statistics** | **Mean(**$\mathbf{MD}$**_Train)** | **Var(**$\mathbf{MD}$**_Train)** | **Mean(**$\mathbf{MD}$**_Test)** | **Var(**$\mathbf{MD}$**_Test)** |
| --- | --- | --- | --- | --- |
| $N=25$ | 32.88 | 1.77 | 216.86 | 2984.0 |
| $N=100$ | 81.65 | 7.49 | 288.58 | 1154.38 |
| $N=1000$ | 229.58 | 317.25 | 297.52 | 878.34 |
| $N=10000$ | 248.30 | 481.31 | 254.56 | 527.49 |

**13. Impact of the decision criteria on the Type I error**

In this simulation we aim to identify which statistical approach is better suited to characterize the $MD$ computed using a shrunken estimator. To this end, we have used the “LW” shrinkage and we have simulated a multivariate normal distribution using $N\in\{25, 50, 100, 200\}$ and $P\in\{250, 500, 1000\}$ for the baseline observations, in order to test the performance of the approaches (Bootstrap- vs $\chi^{2}-$based) for a range of values of $P$ in relation to $N$. Figure 4 illustrates the behaviour of the two different decision criteria for the different combinations of $N$ and $P$, using a type (iii) covariance structure. The statistic based on the $\chi^{2}$ distribution constantly outperforms in all scenarios the non-parametric bootstrap, with a systematically lower type I error. For the upcoming discussions and comparison, we will adopt this criterion to define the acceptance area of the control set.


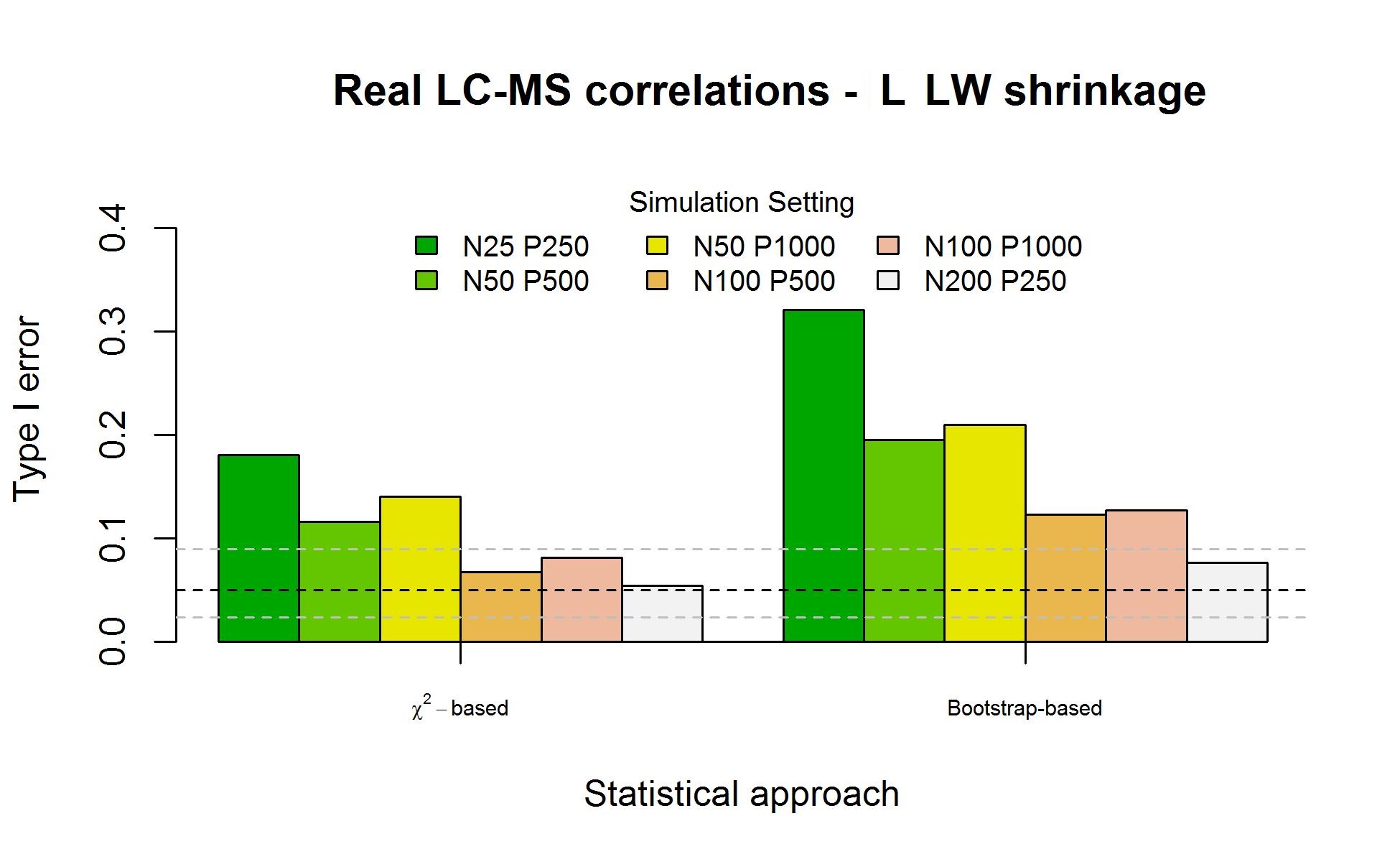


Figure S13: Type I error (over $200$ simulations) for the “L W” eigenvalue shrinkage approaches for the $\mathrm{MD}$ with $\chi^{2}$ or bootstrap-based critical limits for a P-variate normal distribution with $\mathbf{0}$ mean and type (i) covariance matrix ($P=250$). The horizontal black dashed line indicates the expected type I error (0.05) with the respective binomial Clopper-Pearson interval (horizontal grey-dashed line), with parameters $0.05*200$ and $200$. The test data has the same multivariate normal structure of the train data, with a fixed sample size of $N=100$, where $P$ equals the size of the train data.

References

[1] D. I. Warton, “Penalized normal likelihood and ridge regularization of correlation and covariance matrices,” *J. Am. Stat. Assoc.*, vol. 103, no. 481, pp. 340–349, 2008.

[2] D. J. Stekhoven and P. Bühlmann, “MissForest—non-parametric missing value imputation for mixed-type data,” *Bioinformatics*, vol. 28, no. 1, pp. 112–118, Jan. 2012.

[3] J. Engel, L. Blanchet, U. F. H. Engelke, R. A. Wevers, and L. M. C. Buydens, “Towards the disease biomarker in an individual patient using statistical health monitoring,” *PLoS One*, vol. 9, no. 4, 2014.

[4] W. B. Dunn *et al.*, “Molecular phenotyping of a UK population: defining the human serum metabolome,” *Metabolomics*, vol. 11, no. 1, pp. 9–26, 2014.

| 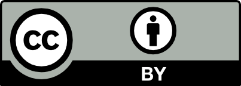 | © 2019 by the authors. Submitted for possible open access publication under the terms and conditions of the Creative Commons Attribution (CC BY) license (http://creativecommons.org/licenses/by/4.0/). |
| --- | --- |
